# Protocol for a randomized controlled trial evaluating the impact of the Nurse-Family Partnership’s home visiting program in South Carolina on maternal and child health outcomes

**DOI:** 10.1186/s13063-020-04916-9

**Published:** 2020-12-04

**Authors:** Margaret A. McConnell, R. Annetta Zhou, Michelle W. Martin, Rebecca A. Gourevitch, Maria Steenland, Mary Ann Bates, Chloe Zera, Michele Hacker, Alyna Chien, Katherine Baicker

**Affiliations:** 1 Department of Global Health and Population, Harvard T.H. Chan School of Public Health, 665 Huntington Avenue, Boston, MA 02115 USA; 2 National Bureau of Economic Research (NBER), 1050 Massachusetts Avenue, Cambridge, MA 02138 USA; 3 Department of Social and Behavioral Sciences, Harvard T.H. Chan School of Public Health, 677 Huntington Avenue, Boston, MA 02115 USA; 4 Department of Health Care Policy, Harvard Medical School, 108 Longwood Avenue, Boston, MA 02115 USA; 5 Population Studies and Training Center, Brown University, Box 1836, 68 Waterman Street, Providence, RI 02912 USA; 6 Abdul Latif Jameel Poverty Action Lab (J-PAL), Massachusetts Institute of Technology, 400 Main Street, E19-201, Cambridge, MA 02142 USA; 7 Division of Maternal Fetal Medicine, Department of Obstetrics and Gynecology, Beth Israel Deaconess Medical Center, 330 Brookline Avenue, Boston, MA 02215 USA; 8 Department of Obstetrics, Gynecology and Reproductive Biology, Harvard Medical School, 260 Longwood Avenue, Boston, MA 02115 USA; 9 Department of Obstetrics and Gynecology, Beth Israel Deaconess Medical Center, 330 Brookline Avenue, Boston, MA 02215 USA; 10 Department of Epidemiology, Harvard T.H. Chan School of Public Health, 677 Huntington Avenue, Boston, MA 02115 USA; 11 Department of Pediatrics, Harvard Medical School, 260 Longwood Avenue, Boston, MA 02115 USA; 12 Department of Medicine, Boston Children’s Hospital, 300 Longwood Avenue, Boston, MA 02115 USA; 13 University of Chicago Harris School of Public Policy, Chicago, IL USA

**Keywords:** Pregnancy, Early childhood, Home visiting, Nurse home visiting, Care coordination, Medicaid, South Carolina, Premature birth, Family planning, Randomized controlled trial

## Abstract

**Background:**

Policy-makers are increasingly seeking rigorous evidence on the impact of programs that go beyond typical health care settings to improve outcomes for low-income families during the critical period around the transition to parenthood and through early childhood.

**Methods:**

This study is a randomized controlled trial evaluating the impact of the Nurse-Family Partnership’s expansion in South Carolina. The scientific trial was made possible by a “Pay for Success” program embedded within a 1915(b) Waiver from Medicaid secured by the South Carolina Department of Health and Human Services. This protocol describes study procedures and defines primary and secondary health-related outcomes that can be observed during the intervention period (including pregnancy through the child’s first 2 years of life). Primary study outcomes include (1) a composite indicator for adverse birth outcomes including being born small for gestational age, low birth weight (less than 2500 g), preterm birth (less than 37 weeks’ gestation), or perinatal mortality (fetal death at or after 20 weeks of gestation or mortality in the first 7 days of life), (2) a composite outcome indicating health care utilization or mortality associated with major injury or concern for abuse or neglect occurring during the child’s first 24 months of life, and (3) an indicator for an inter-birth interval of < 21 months. Secondary outcomes are defined similarly in three domains: (1) improving pregnancy and birth outcomes, (2) improving child health and development, and (3) altering the maternal life course through changes in family planning.

**Discussion:**

Evidence from this trial on the impact of home visiting services delivered at scale as part of a Medicaid benefit can provide policy-makers and stakeholders with crucial information about the effectiveness of home visiting programs in improving health and well-being for low-income mothers and children and about novel financing mechanisms for cross-silo interventions.

**Trial registration:**

The trial was registered prospectively on the American Economic Association Trial Registry (the primary registry for academic economists doing policy trials) on 16 February 2016 (AEARCTR-0001039). ClinicalTrials.gov NCT03360539. Registered on 28 November 2017.

## Administrative information

**Table:** 

**Data category**	**Information**
Title	Protocol for a randomized controlled trial evaluating the impact of the Nurse-Family Partnership’s home visiting program in South Carolina on maternal and child health outcomes
Trial Registration:	AEA RCT Registry: AEARCTR-0001039 Clinicaltrials.gov: NCT03360539
Primary registry and trial identifying number	AEA RCT Registry: AEARCTR-0001039
Date of registration in primary registry	February 20, 2016
Secondary identifying numbers	ClinicalTrials.gov: NCT03360539
Source(s) of monetary or material support	Children’s Trust of South Carolina, Arnold Ventures, The Duke Endowment, The BlueCross BlueShield Foundation of South Carolina
Primary sponsor	Harvard T.H. Chan School of Public Health
Secondary sponsor(s)	Abdul Latif Jameel Poverty Action Lab; University of Chicago
Contact for public queries	Margaret McConnell mmcconne@hsph.harvard.edu
Contact for scientific queries	Margaret McConnell mmcconne@hsph.harvard.edu
Public title	Nurse-Family Partnership Impact Evaluation in South Carolina (NFP)
Scientific title	Protocol for a randomized controlled trial evaluating the impact of the Nurse-Family Partnership’s home visiting program in South Carolina on maternal and child health outcomes
Countries of recruitment	United States of America
Health condition(s) or problem(s) studied	Preterm Birth; Injuries; Maternal Behavior
Intervention(s)	Experimental: Treatment—Nurse-Family Partnership prenatal and infancy home visiting program providing with regular visits to first-time mothers until the child is two years old
	No intervention: Control group members have access to the standard of care
Key inclusion and exclusion criteria	Ages Eligible for Study: 15–55 Years; Sexes Eligible for Study: Female; Accepts Healthy Volunteers: Yes
	Inclusion Criteria: Female; No previous live births; Currently pregnant; Gestation period less than 28 weeks (i.e., less than or equal to 27 weeks, 6 days) at time of recruitment; Ages 15–55; Income level meets Medicaid eligibility criteria; Live within an area serviced by a NFP Implementing Agency; Not currently enrolled in the study; Not incarcerated or living in lock down facilities
	Exclusion Criteria: Men; Women who have had a previous live birth; Women who are not currently pregnant; Women who are past their 28th week of gestation (i.e., greater than or equal to 28 weeks, 0 days) at time of recruitment; Women who are younger than 15 or older than 55 years of age; Women whose income level does not meet Medicaid eligibility criteria; Women who live outside of an area serviced by a NFP Implementing Agency; Women who are currently enrolled in the study; Women who are currently incarcerated or living in a lock down facility
Study type	Allocation: Randomized Intervention model: Single Group Assignment Masking: None (Open Label) Primary purpose: Supportive Care
Date of first enrolment	April 1, 2016
Target sample size	6000 participants
Recruitment status	Closed
Primary outcome(s)	Composite of Small for gestational age, or Low birth weight (less than 2500 g), or Preterm Birth (less than 37 weeks’ gestation by obstetric estimate) or Perinatal Mortality (fetal death at or after 20 weeks of gestation or mortality within first 7 days of life) [Time Frame: Captured by birth certificates at birth (Jan 2021)] Composite of either major injury or concern for abuse or neglect [Time Frame: Captured by Medicaid claims, hospital discharge at 24 months postpartum] Inter-birth interval of < 21 months [Time Frame: Captured by birth certificates at 21 months (October 2022)]
Key secondary outcomes	Small for gestational age [Time Frame: Captured by birth certificates at 0 months (Jan 2021)] (See Table 1 for complete list of secondary outcomes)
Protocol version	Issue date: January 30, 2020 Protocol amendment number: 02 Authors: MAM,^1^ AZ,^2^ MWM,^3^ RG,^4^ MS,^5^ MB,^6^ CZ,^7,9^ MH,^8,9,10^ AC,^11,12^ KB^2,6,13^ Revision chronology: August 19, 2015—Original Draft January 30, 2020—Draft for first journal submission October 21, 2020—Draft responding to reviewer comments
Author details	Authors: MM,^1^ AZ,^2^ MWM,^3^ RG,^4^ MS,^5^ MB,^6^ CZ,^7,9^ MH,^8,9,10^ AC,^11,12^ KB^2,13^ ^1^ Department of Global Health and Population, Harvard T.H. Chan School of Public Health, Boston, MA ^2^ National Bureau of Economic Research (NBER), Cambridge, MA ^3^ Department of Social and Behavioral Sciences, Harvard T.H. Chan School of Public Health, Boston, MA ^4^ Department of Health Care Policy, Harvard Medical School, Boston, MA ^5^ Populations Studies and Training Center, Brown University, Providence, RI ^6^ Abdul Latif Jameel Poverty Action Lab (J-PAL), Cambridge, MA ^7^ Division of Maternal Fetal Medicine, Department of Obstetrics and Gynecology, Beth Israel Deaconess Medical Center, Boston, MA ^8^ Department of Obstetrics and Gynecology, Beth Israel Deaconess Medical Center, Boston, MA ^9^ Department of Obstetrics, Gynecology and Reproductive Biology, Harvard Medical School, Boston, MA ^10^ Department of Epidemiology, Harvard T.H. Chan School of Public Health, Boston, MA ^11^ Department of Pediatrics, Harvard Medical School, Harvard University, Boston, MA ^12^ Department of Medicine, Boston Children’s Hospital, Boston, MA ^13^ University of Chicago Harris School of Public Policy, Chicago, IL Authors’ contributions: AZ, MWM, MB, KB: Conception and development MAM, AZ, MWM, MB, KB: Study design MAM, MWM, RG, MS, CZ, MH, AC: Design of outcomes MAM: Original Draft of manuscript MAM, AZ, MWM, RG, MS, MB, CZ, MH, AC, KB: Editing and review of manuscript and approval of final version
Trial sponsor	Trial Sponsor: Harvard T.H. Chan School of Public Health Sponsor’s Reference: FWA00002642 Contact name: Margaret McConnell, PhD Address: 677 Huntington Avenue, Boston Massachusetts Telephone: (203)745-8321 Email: mmcconne@hsph.harvard.edu
Role of study sponsor and funders	The research team received feedback on the proposed research from the Pay for Success (PFS) contract signatories (including the Nurse-Family Partnership) and the funders that informed the approach to outcome selection and the definition of subgroups. PFS signatories and funders will have no influence on the analysis of data or reporting of results.
Other roles	The Abdul Latif Jameel Poverty Action Lab has led the implementation of the trial. Sam Ayers, Adam Baybutt, Kim Gannon, Noreen Giga, Elisabeth O’Toole and Pauline Shoemaker all contributed substantially to the development and implementation of the trial.

We present the full SPIRIT Checklist in Additional file 1.

## Background

### Addressing the challenges of childhood poverty with community-based medicine

Millions of children in the USA live in households experiencing poverty [1], and nearly half of children born into poverty stay there for the remainder of their childhood [2]. Childhood poverty is linked to adverse children’s health and development outcomes, and early life experiences can shape children’s long-term outcomes well into adulthood [3–5]. There is growing policy interest in addressing the challenges faced by low-income families during early childhood, with a recognition that effective policies and interventions will address maternal and child well-being in tandem [6]. Policy-makers increasingly seek to address the social determinants that may contribute to poor health outcomes for low-income mothers and young children, such as access to food and housing, environmental factors, and economic disadvantage [7, 8]. Moreover, there is an acknowledgement that traditional clinical settings may not be well-suited to meeting these challenges [9]. As a result, some policy-makers administering Medicaid are seeking to support programs that can reach beyond the capability of the traditional clinical approach to pregnancy, childbirth, and medical care [10–17], to more comprehensively address the sources of disparities that are already entrenched by the time children enter kindergarten [18, 19].

### Nurse-Family Partnership model

One of the established models for reaching low-income families to address a range of challenges in early life is nurse home visiting during pregnancy and early childhood. From 1978 to 1995, three modest-scale randomized controlled trials were conducted in Elmira, New York; Memphis, Tennessee; and Denver, Colorado, to estimate the impact of the Nurse-Family Partnership (NFP) on the outcomes of low-income families over multiple decades [20–22]. The evidence from these randomized controlled trials played a key role in the expansion of millions of dollars in philanthropic, local, state, and federal funding, including for home visiting services through the U.S. Department of Health and Human Services’ Maternal, Infant, and Early Childhood Home Visiting (MIECHV) program [23].

### Context of the South Carolina trial

The extent to which evidence from these early trials of NFP applies within a modern context is an important policy question. Over the past decades, the health care and social safety net landscapes have changed substantially, as have the composition, health status, and experiences of low-income families. The scale of NFP has also increased substantially; earlier trials represent the impact of the program on a small scale within narrowly defined populations. Earlier trials were also conducted before current practices in pre-registration of outcomes for clinical trials and accounting for multiple inferences were developed. Recent evidence on the impact of similar nurse-led home visiting programs has been mixed [24, 25], and policy-makers want to understand whether scaling home visiting through public insurance can improve maternal and child well-being at the population level [26].

Our ongoing experiment in South Carolina represents a rare opportunity to understand the effects of NFP when scaled up to serve a broader population in today’s context. The South Carolina Department of Health and Human Services (SCDHHS) is offering NFP services to first-time, Medicaid-eligible mothers by leveraging Medicaid funding via a Medicaid 1915(b) Waiver and philanthropic funding. The financing operates through a Pay for Success (PFS) model, where program costs are initially covered via philanthropic and Medicaid funding, and later SCDHHS will make success payments if our randomized controlled trial provides impact estimates that exceed the impact thresholds that were defined in the PFS contract before the trial began [27].

### Protocol paper focus: maternal and child health outcomes during pregnancy and early childhood

Home visiting programs have been hypothesized to affect a diverse range of outcomes, including prenatal, maternal, and neonatal health, infant and child health and development, morbidity and mortality, mental health, substance abuse, family planning, nutrition, neglect and maltreatment, home environment and parenting skills, crime, educational attainment, public spending, family economic self-sufficiency, access to healthcare, community resource connections, and social competence [28]. This protocol paper focuses on defining health-related outcomes that may be observed during the period of pregnancy and through the child’s first 2 years of life, when the family would be eligible for nurse home visiting services through NFP. We will assess outcomes across a broader group of domains and longer time horizon in future work.

Low-income families face particular challenges and disadvantages during the critical period around pregnancy, birth, transition to parenthood, and early childhood. These include relatively worse access to and quality of health care [29, 30] and increased exposure to environmental, neighborhood, or housing-related risks [8, 31–34]. The health risks associated with pregnancy, childbirth, and the postpartum period are significant, particularly for low-income households. Maternal mortality rates have doubled in the USA over the last two decades [35], and preterm birth rates have risen each year over the last 3 years [36]. Substantial racial disparities have been documented surrounding childbirth, including higher rates of maternal mortality and morbidity and a higher likelihood of babies born preterm and low birth weight for black women compared to non-Hispanic white women [37–39]. Geographic disparities have also been documented; women living in rural areas are more likely to experience preterm birth, infant mortality, or maternal mortality than those living in urban areas [40, 41], and are more likely to face the closure of their nearest hospital [40]. Unintended and short-interval births are associated with adverse health and social consequences and are also substantially more likely for low-income families [42]. Pregnancy intervals of less than 6 months are associated with adverse newborn health outcomes including low birth weight, small for gestational age and infant mortality [43]. Both children whose birth occurred earlier than desired and their siblings receive substantially less maternal investment than those born at the desired time [44]. Substantial evidence suggests better access to contraception that allows for achieving desired timing of births can improve maternal educational achievement and economic outcomes [45] with corresponding reductions in child poverty [46]. Finally, the direct effects of the strain of experiencing poverty itself can limit adults’ attentional and cognitive resources [47–50] available for parenting. Children from low-income families and communities with high rates of poverty are substantially more likely to experience adverse events including abuse or neglect [33, 51, 52], which is associated with long-term impacts on adult health [53, 54] and well-being [1, 55]. The leading cause of death of children under five is unintentional injury, which is more common in low-income neighborhoods [56]. Major unintentional child injuries are also more likely to occur in households of lower socioeconomic status [57, 58]. In this paper, we describe scientific outcomes which are designed to capture the potential impact of home visiting programs within the context of these challenges.

## Methods

This study is an individually randomized controlled parallel group trial.

### Ethical considerations

The study was approved by the Harvard T.H. Chan School of Public Health Institutional Review Board (IRB15-2939). Permissions were also obtained from cooperating institutions. The following IRBs have approved Harvard’s oversight of this research study: Massachusetts Institute of Technology, South Carolina Department of Health and Environmental Control (DHEC), IRB00000092 (which includes DHEC agencies covering the Upstate, Pee Dee, Lowcountry and Midlands regions), McLeod Health System, IRB00004313, Greenville Health System, and Spartanburg Regional Health System, IRB00001369.

### Intervention: Nurse-Family Partnership program

NFP is a prenatal and infancy home visiting program for low-income, first-time mothers, and their families. Registered nurses enroll pregnant women who have completed less than 28 weeks’ gestation. NFP attempts to enroll women early in pregnancy so that home visits can take place throughout the pregnancy. This enables nurse home visitors to monitor pregnancy health at home, encourage utilization of high-quality health care during pregnancy, and help pregnant women make informed choices about their own health and the health of their baby. Nurses continue regular visits with the family until the child is 2 years old. Families may choose to discontinue participation in nurse home visiting services at any point. During the study period, mothers enrolled in the NFP program were eligible for up to 40 home visits divided into up to 15 visits during the prenatal period, up to 8 visits during the postpartum period (within the 60 days after delivery), and up to 17 visits during the child’s first 24 months of life that were covered by the Medicaid waiver (described below). When convenient for the mother, the nurses could conduct telehealth visits instead of doing visits in the home. Services are provided in English and Spanish and translation services are available for mothers speaking other languages.

### Pay for success background

With a limited Medicaid budget and the desire to improve early childhood outcomes statewide, SCDHHS sought to expand NFP to eligible Medicaid recipients by applying for a 1915(b) Waiver and establishing a “Pay for Success” (PFS) Contract. The Medicaid Waiver authorized South Carolina to expand its current postpartum home visitation services by scaling up NFP. Under the Waiver, an additional 3200 Medicaid beneficiaries and their children were made eligible to enroll in NFP services during the study enrollment period (between April 1, 2016, and March 31, 2020). As part of the Waiver, SCDHHS established a PFS contract with a consortium of local philanthropic agencies to secure the additional funding needed to scale up the program over the 5-year Waiver period. The philanthropic agencies provide the initial capital to scale up NFP (in combination with federal dollars from MIECHV and the Medicaid Waiver); based on point estimates of the program’s impact on four pre-determined outcomes, SCDHHS will make “success payments” back to the funders, who have agreed to reinvest the money to expand NFP in South Carolina in subsequent years. The four outcomes on which payments will be made are preterm birth, child injury, birth spacing, and the percent of served mothers who live in low-income zip codes. Additional file 2 describes the PFS outcomes and thresholds for payments. The signatories of the contract are SCDHHS, NFP, and the Children’s Trust of South Carolina (which administers funds from the philanthropic partners, MIECHV, and potential success payments). As independent evaluators, the research team was not a signatory of the PFS contract.

The first payments are scheduled to be made in April 2021 for all four study outcomes. Payment outcomes were chosen by the signatories to the contract to reflect state-level priorities and major drivers of Medicaid spending. This protocol paper describes health-related primary and secondary outcomes chosen by the research team which are informed by, but distinct from, the outcomes chosen by signatories to the PFS contract.

### NFP program implementation in South Carolina

**Fig. 1:**
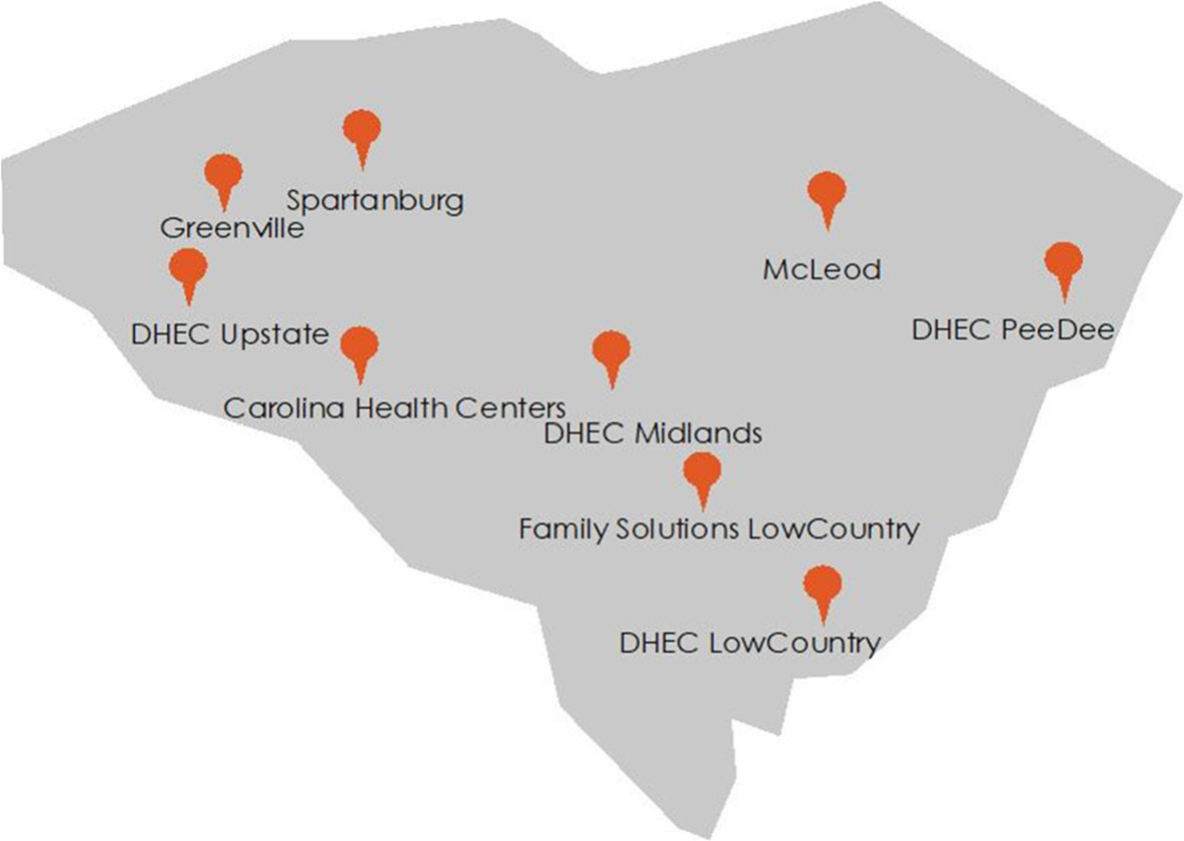
NFP sites across South Carolina

NFP has a well-established delivery system in South Carolina, and there are multiple pathways through which potential clients can be referred. NFP has operated in South Carolina since 2009 and is delivered by 10 different implementing agencies in 32 counties across the state. A map of NFP implementing agency locations in South Carolina is provided in Fig. 1. The catchment area covers both urban and rural South Carolina. Nine of the implementing agencies participated in this project, which represented a significant scale-up of NFP services throughout the state. Prior to the launch of the study, NFP served approximately 500–600 moms annually across the state. During the study enrollment, NFP scaled up to serve an average of 1200 women each year with a corresponding increase in staffing of nurse home visitors and supervisors.

### Eligibility criteria

Individuals are eligible for the study if they (1) are currently pregnant with a gestation period less than 28 weeks, (2) would be first-time mothers, (3) would be income-eligible for Medicaid during pregnancy, (4) are at least 15 years old, (5) live in a catchment area served by NFP nurses, (6) are not incarcerated or living in a lockdown facility, and (7) have enough language fluency that they would be able to benefit therapeutically from the program.

### Referral

Potential study participants are identified through several referral channels. First, referral partners, such as local health care providers, schools, and Special Supplemental Nutrition Program for Women, Infants, and Children (WIC) agencies, directly refer potential clients to an implementing agency with the client’s permission. Second, referrals are made directly from the Medicaid eligibility database to NFP. Finally, during the scale-up of NFP services, NFP had in place an outreach team with outreach coordinators in four regional areas who worked to identify potential clients. In addition to these main channels, some clients are self-referred or referred by a family member or friend or through digital and print advertisements of the program throughout South Carolina.

### Enrollment and informed consent

Potential participants are assessed first for eligibility by NFP nurses and then provide written consent to participate in the study. NFP program staff are trained on how to implement the informed consent process by the study team. To minimize coercion and undue influence, program staff review the consent form with potential participants and allow them as much time as they need to read the consent form themselves and ask questions. The consent form (provided in Additional file 3) informs participants about randomization and participation in NFP and informs clients that researchers will track their data and their children’s data across a variety of administrative data records for up to 30 years. Electronic signatures are obtained from consenting study participants.

### Randomization

**Fig. 2:**
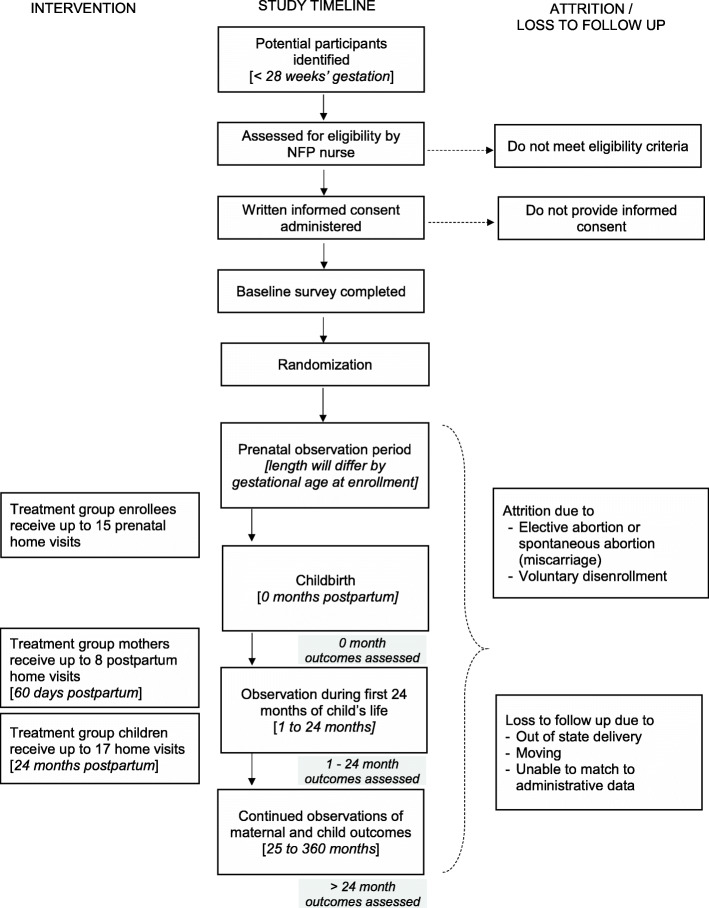
Study timeline

This study is a parallel group individually randomized controlled trial. Participant flow through study procedures is provided in Fig. 2. Study participants and program delivery staff are (perforce) not blinded to treatment-control status. After providing informed consent and completing a baseline survey, mothers are randomly assigned either to a treatment group that is offered access to NFP or to a control group that has access to the standard of care and other available community programs and services, but not NFP. Two thirds of the participants are randomly assigned to the treatment group and one third to the control group. This assignment ratio maximizes the use of existing resources while maintaining adequate statistical power.

Individuals assigned to the treatment group participate in typical NFP program activities. Individuals randomized into the control group receive the standard of care in South Carolina. They are not offered participation in NFP, but may receive all community and medical services to which they would otherwise be entitled, including up to two postpartum home visits routinely paid for by Medicaid in South Carolina. Both control and treatment group enrollees in the study are provided with a list of community resources available to low-income new mothers (sample list provided in Additional file 4).

Study enrollment, on-the-spot randomization, and the baseline survey are conducted by NFP staff using encrypted tablets and computer-assisted personal interview (CAPI) software. We use SurveyCTO, which provides full customization of the baseline survey described in the Baseline survey section, real-time randomization, built-in time-stamping, and audio-recording capabilities to monitor fielding quality and fidelity to protocols. The enrollment software automatically checks identifying information provided during the baseline survey to ensure that no individual can enroll in the study more than once, avoiding potential gaming of the random assignment mechanism.

### Data sources

#### Baseline survey

Prior to randomization, study participants complete a 30-min baseline survey (see Additional file 5) covering demographics, physical and mental health, health behaviors, care-seeking, use of social services, socioeconomic status, and their relationship with the father of the child. Mothers also provide identifying information such as social security number, birthdate, and other information that can be used to match the mother to outcomes in administrative data records. Each mother receives a $25 gift card as compensation for her time completing the baseline survey. Data collected at baseline is used to describe the characteristics of the study sample, link to administrative records, assess the baseline equivalence of the treatment and control groups at the point of randomization, and provide baseline covariates for the impact models.

#### Survey of nurses

We also conducted a survey of nurse home visitors who have delivered home visiting services to mothers enrolled during the trial. The survey was administered prior to the end of study enrollment. The survey (see Additional file 6) covered nurse demographics, work history, nurse home visiting practices and workload, referral patterns, and perceptions of potential impact on mothers and children. The survey will provide contextual background and help evaluate any heterogeneity in treatment impacts.

#### Administrative data sources and matching to outcomes

All study outcomes will come from linking participants to outcomes observed in administrative data. The informed consent process provides access to a broad range of administrative data on the health and well-being of mothers and their children in both treatment and control arms for up to 30 years. South Carolina is a particularly data-rich state: there is an agency dedicated to linking an extensive range of administrative data—from health care to social services to criminal justice to employment and beyond—with a track record of linking and securely providing data to researchers. We have secured data use agreements with multiple agencies, including for example the South Carolina Revenue and Fiscal Affairs Office, Departments of Health and Environmental Control, Social Services, Education, Mental Health, and Law Enforcement and Corrections. Furthermore, we have a data use agreement with NFP that allows us to follow the mother’s participation in NFP program activities (including whether they participate in the recommended frequency of home visiting services) and a data use agreement with the Children’s Trust Foundation of South Carolina which allows us to track the participation of mothers enrolled in the study in other home visiting programs.

We will match mothers enrolled in our study to administrative outcome data using a probabilistic match based on identifying information provided during the baseline survey (including social security number, birth date, name, and Medicaid ID). We will identify children born into the study by first matching the mother to a birth in vital records. If the birth occurs within 120 days before or after the estimated due date reported on the baseline survey, we will consider the birth as being related to the pregnancy that was in gestation at the time of the baseline survey. Births that occur outside of this window will be considered unmatched (potentially due to a miscarriage followed by a subsequent birth) and will not be included in our analysis.

### NFP theory of change and program content

NFP’s program strives to impact the lives of mothers and children in three central ways: (1) to improve pregnancy outcomes, (2) to improve child health and development, and (3) to improve economic self-sufficiency. NFP aims to achieve these outcomes through a strength-based approach—which is based on the idea that new mothers will be best able to make changes in their lives when building on their own knowledge and strengths [59]. NFP home visiting services are also delivered through a therapeutic, relationship-based model where nurse home visitors typically form long-term, trusting relationships with mothers [59]. Activities to achieve these outcomes center around five main domains: (a) maternal health, (b) the home environment, (c) maternal life course development or goals for the future, (d) mothers’ role in the child’s health and development, and (e) strategies for leveraging social support. Home visitors seek input from clients in choosing which areas to prioritize by routinely assessing mothers’ physical and mental health and social determinants of health (e.g., unsafe housing, food insecurity, or social isolation) to identify the mother’s primary concerns. Nurse home visitors utilize “facilitators”—a guided tool with pre-programmed content—to educate mothers and motivational interviewing techniques to empower mothers to advocate for themselves. Nurse home visitors also provide referrals and care coordination to needed health care providers and community resources. They may also perform monitoring services such as taking maternal weight or blood pressure. In many (but not all) implementing agencies, nurse home visitors have access to electronic medical records and may be able to access clinical information about patient’s health during pregnancy and postpartum.

### Existing evidence base on NFP services

NFP’s focus on maternal and newborn health is consistent with the focus of the federal MIECHV program (funded by the Health Resources and Services Administration) [60, 61] which lists maternal and newborn health as the first central objective of home visiting programs. Unlike many home visiting programs which target families after birth, NFP delivers home visiting services throughout pregnancy, maximizing the potential to influence maternal and newborn outcomes. Evidence on the impact of home visiting programs on maternal and newborn health is mixed. An early trial evaluating the impact of NFP in Elmira, NY, found that adolescent mothers randomly assigned to receive NFP services had babies with higher birth weights, and mothers in the treatment group who reported smoking during pregnancy experienced a reduced likelihood of preterm birth [62]. Another early evaluation of NFP in Memphis, TN, found that mothers assigned to receive NFP services were less likely to experience hypertensive disorders of pregnancy, but had no reduced likelihood of preterm birth or low birth weight [22]. More recently, a large-scale randomized trial conducted to evaluate the impact of the MIECHV program [63] and an evaluation of a home visiting program in the UK found no evidence of impacts on birth outcomes [24].

Second, home visiting programs, because they focus on improving the safety of the home environment and providing parents with a broad set of tools and resources to help them be more effective parents, have sought to reduce the risk of child injuries and indicators of child abuse and neglect. Home visiting programs have been cited as one of the few interventions where rigorous evidence has demonstrated the potential to improve child outcomes surrounding child maltreatment [55]. An early randomized trial evaluating the impact of the NFP program delivered in Memphis, TN, found 22% fewer health care encounters for child injuries and ingestions and a 78% reduction in days hospitalized for injuries or ingestions [22].

Finally, home visiting programs may alter maternal life course and economic opportunity through a variety of channels with the potential for these alterations to change the timing of subsequent births. Home visiting programs often serve a population of first-time low-income mothers who have greater likelihood of unplanned or mis-timed births and who may desire to avoid or delay subsequent pregnancies. Indeed, among the 5655 study participants, 82.7% reported on the baseline survey that their pregnancies were either unplanned or occurred earlier than the mother desired, and only 3.3% of mothers reported desiring less than 2 years of spacing between this birth and any subsequent birth. Home visiting programs may be effective in increasing birth spacing by coordinating continuity of care and ensuring that postpartum mothers have access to family planning services. Home visiting programs also focus on enabling mothers to alter their life course by taking advantage of educational and career opportunities, which may delay subsequent pregnancies. Evidence from previous evaluations of NFP have found substantial impacts on shaping the maternal life course by altering the patterns of subsequent births. In the early evaluation of NFP in Elmira, New York, mothers reported 19% fewer subsequent births by the time their first child reached the age of 15 [64]. In the trial of NFP conducted in Memphis, Tennessee, a subset of the sample consisting of adolescent mothers reported 16% fewer births and a significant increase in birth intervals between their first and second child at a 6-year follow-up [65].

### Primary study outcomes

We define three primary outcomes for this analysis, one for each of the three domains that home visiting programs, including NFP, present as central program objectives or areas where home visiting programs have demonstrated substantial health-related impacts. In addition to these three primary outcomes, we provide a list of pre-specified secondary outcomes in each of these domains in Table 1. We define each outcome with the mother as the unit of observation (instead of considering outcomes separately for multiple births). In future work, we plan to explore NFP’s impact on other domains and over longer time horizons.

**Table 1:** NFP program objectives and corresponding primary and secondary outcomes

	Data source(s)	Time at complete outcome observation (time since all pregnancies completed)
**Objective 1. Improve pregnancy, birth, and maternal health outcomes**		
Primary outcome		
Composite of at least one of: • Small for gestational age, or • Low birth weight (less than 2500 g), or • Preterm birth (less than 37 weeks’ gestation by obstetric estimate), or • Perinatal mortality (fetal death at or after 20 weeks of gestation or mortality in the first 7 days of life)	Birth certificates, fetal death records, mortality records	1 months (Feb 2021)
Secondary outcomes		
*Infant outcomes observed at birth*		
Small for gestational age	Birth certificates	0 months (Jan 2021)
Large for gestational age	Birth certificates	0 months (Jan 2021)
Low birth weight (< 2500 g)	Birth certificates	0 months (Jan 2021)
Very low birth weight (< 1500 g)	Birth certificates	0 months (Jan 2021)
Birth weight (continuous)	Birth certificates	0 months (Jan 2021)
Preterm birth (< 37 weeks’ gestation by obstetric estimate)^1^	Birth certificates	0 months (Jan 2021)
Extremely preterm (< 28 weeks’ gestation)	Birth certificates	0 months (Jan 2021)
Gestational age at birth in weeks (continuous)	Birth certificates	0 months (Jan 2021)
Perinatal mortality (fetal death at or after 20 weeks of gestation or mortality in the first 7 days of life)	Fetal death records, mortality records	1 months (Feb 2021)
NICU admission of at least overnight	Hospital discharge	0 months (Jan 2021)
Neonatal morbidity^2^	Hospital discharge	0 months (Jan 2021)
*Maternal outcomes*		
Cesarean delivery	Birth certificates	0 months (Jan 2021)
Severe acute maternal morbidity^3^	Hospital discharge	0 months (Jan 2021)
Maternal mortality (up to 1 year after birth)	Mortality records	12 months (Jan 2022)
Neonatal abstinence disorder or maternal drug/substance abuse	Medicaid claims, hospital discharge	24 months (January 2023)
Maternal experience of violence or homicide^4^	Medicaid claims, mortality records	24 months (January 2023)
Postpartum visit within the first 12 weeks postpartum	Medicaid claims	2 months (March 2021)
*Utilization and quality of prenatal care*		
Adequate prenatal care (Adequacy of Prenatal Care Utilization (APNCU) Index)	Birth certificates	0 months (Jan 2021)
Number of emergency department visits during pregnancy	Hospital discharge	0 months (Jan 2021)
Dental visit (preventive or treatment) during pregnancy	Medicaid medical and dental claims	0 months (Jan 2021)
Ultrasound at 18–22 weeks (anatomy scan)	Medicaid claims	0 months (Jan 2021)
Proportion of recommended prenatal screenings completed^5^	Medicaid claims	0 months (Jan 2021)
*Mental health outcomes*		
Any outpatient treatment or diagnosis^6,7^	Medicaid claims	2 months (March 2021)
Diagnosis of depression/anxiety/stress reaction^6^	Medicaid claims	2 months (March 2021)
Antidepressant prescription^6^	Medicaid claims	2 months (March 2021)
Outpatient mental health visit^6^	Medicaid claims	2 months (March 2021)
Treatment follow-up^8^	Medicaid claims	6 months (July 2021)
Mental health-related emergency/inpatient visit^9^	Hospital discharge	12 months (January 2022)
Number of mental health-related emergency/impatient visits^9^	Hospital discharge	12 months (January 2022)
**Objective 2. Improve child health and development**		
Primary outcome		
Composite of at least one health care encounter or mortality associated with ICD codes indicating at least one of the following: • Major injury, or • Concern for abuse or neglect	Medicaid claims, hospital discharge, mortality files (defined in Tables 2 and 3)	24 months (January 2023)
Secondary outcomes		
*Accidents, suspected abuse and neglect and emergency care utilization*		
Health care encounter or mortality associated with ICD codes indicating major injury	Medicaid claims, hospital discharge, mortality files (defined in Table 2)	24 months (January 2023)
Health care encounter or mortality associated with ICD codes indicating concern for abuse or neglect	Medicaid claims, hospital discharge, mortality files (defined in Table 3)	24 months (January 2023)
Number of injuries^10^	Hospital discharge	24 months (February 2023)
Any emergency department visit	Hospital discharge	24 months (January 2023)
Number of emergency department visits	Hospital discharge	24 months (January 2023)
All-cause child mortality in first 24 months of life or fetal death	Fetal death records, mortality records	24 months (January 2023)
*Outcomes related to preventative care*		
Proportion of recommended well-child visits	Medicaid claims	15 months (April 2022)
At least one lead screening	Medicaid claims	15 months (April 2022)
At least one developmental screening^11^	Medicaid claims	12 months (January 2022)
At least one dental visit^12^	Medicaid medical and dental claims	24 months (January 2023)
Share of recommended fluoride treatments^13^	Medicaid medical and dental claims	24 months (January 2023)
**Objective 3. Alter maternal life course**		
Primary outcome		
Inter-birth interval of < 21 months	Birth certificates	21 months (October 2022)
Secondary outcomes		
*Birth spacing outcomes*		
Inter-birth interval of < 24 months^14^	Birth certificates	24 months (January 2023)
Inter-birth interval of < 15 months	Birth certificates	15 months (April 2022)
Inter-birth interval (continuous)	Birth certificates	60 months (January 2026)
*Postpartum family planning while enrolled in postpartum Medicaid coverage (6 weeks)*		
Any family planning related counseling or service	Medicaid claims, hospital discharge^15^	6 weeks (March 2021)
Received a highly or moderately effective method of contraception^16^	Medicaid claims, hospital discharge	6 weeks (March 2021)
Immediate postpartum long-acting reversible contraception	Medicaid claims, hospital discharge	6 weeks (March 2021)
*Postpartum family planning within 1 year*		
Any family planning related counseling or service	Medicaid claims, hospital discharge	12 months (January 2022)
Received a highly or moderately effective method of contraception^16^	Medicaid claims, hospital discharge	12 months (January 2022)
Postpartum intrauterine device insertion	Medicaid claims, hospital discharge	12 months (January 2022)
*Timing of postpartum family planning take-up*		
Time to first family planning counseling or service (months from pregnancy)	Medicaid claims, hospital discharge	24 months (January 2023)
Time to first utilization of highly effective contraceptive methods (months from discharge)	Medicaid claims, hospital discharge	24 months (January 2023)

^1^Outcome included in Pay for Success contract

^2^Assisted ventilation immediately after delivery, assisted ventilation for more than 6 h, seizure, receipt of surfactant replacement therapy, and receipt of antibiotics for suspected sepsis

^3^As defined by the Centers for Disease Control and Prevention (CDC)

^4^Includes any ICD code for healthcare encounter associated with experiencing violence (codes related to intimate partner violence adapted from Schafer et al. 2008) or mortality associated with homicide based on ICD code

^5^Obstetric panel (D (Rhesus), red blood cell antibody screen, complete blood count, urine culture, urinalysis), Sexually Transmitted Infection screenings (HIV, Syphilis, Hepatitis B, Chlamydia if age < 25 years, gonorrhea if age < 25 years), Group B screening, Glucose screening at 24–28 weeks

^6^During pregnancy or 60 days postpartum

^7^Diagnosis for depression/anxiety/stress reaction or antidepressant prescription or outpatient mental health visit

^8^Second antidepressant prescription or outpatient mental health visit within 120 days of treatment initiation (“acute phase”)

^9^During pregnancy or 12 months postpartum; based on all-listed diagnoses (i.e., primary or secondary) for depression/anxiety/stress reaction

^10^Based on ICD codes designated in the Pay for Success (PFS) contract. Outcome included in PFS contract

^11^Recommended at 9 months

^12^Recommended by American Academy of Pediatrics (AAP) / American Academy of Pediatric Dentistry (AAP/AAPD)

^13^AAP/AAPD recommends 4 treatments (one every 6 months). These are covered by Medicaid

^14^Outcome included in Pay for Success (PFS) contract

^15^National Drug Codes/ICD codes derived from OPA and supplemented by ICD any codes indicating family planning counseling

^16^CDC defines highly effective contraception to include implant, immediate postpartum long-acting reversible contraception, long-acting reversible contraception, or sterilization and moderately effective contraception to include path, ring, diaphragm, injectables, and contraceptive pills

First, we will assess the effect of NFP on the likelihood of having an adverse birth outcome. We define an adverse birth outcome as having a preterm birth (less than 37 weeks’ gestation) or a newborn being small for gestational age (less than 10th percentile of US births conditional on gestational age based on specifications provided by Talge et al. [66]), having low birth weight (less than 2500 g) or experiencing perinatal mortality (fetal death at or after 20 weeks of gestation or mortality in the first 7 days of life). Data for the adverse birth outcome will come from South Carolina birth certificates, fetal death, and mortality records. For mothers with multiple births, we define the outcome based on having any adverse birth outcome for any child. While we expect preterm birth and other adverse birth outcomes to occur more commonly among multiple births, we anticipate that rates of multiple births will be balanced across treatment and control arms. We will explore alternative specifications of this outcome that include only singleton births. While the composite includes outcomes with different severity, which may increase the difficulty of interpretation [67], it captures the efforts of NFP nurse home visitors to influence pregnancy outcomes through a variety of channels from direct provision of medical care to increased utilization of clinical services to generalized improvements in general well-being that may translate to reduced stress and anxiety during pregnancy. Our primary outcome capturing the objective to improve pregnancy outcomes focuses on the health of the infant, without considering the health of the mother. We designed this composite outcome in order to maintain a parsimonious list of primary outcomes and to ensure adequate statistical power. Secondary analyses (Table 1) will consider maternal outcomes that occur with lower frequency (including maternal morbidities, mortality, and maternal experiences of violence [68]). We have also planned detailed analyses that will allow for a better understanding of the changes in utilization that result from participation in NFP including antenatal care and mental health care utilization.

Second, we will assess the effect of NFP on the likelihood of experiencing injury, abuse, or neglect during early childhood. This will be defined by a composite outcome indicating a health care encounter or mortality associated with International Classification of Diseases (ICD) codes indicating either a major child injury or suspicion of abuse or neglect. We will identify children who have at least one health care encounter or experience mortality associated with an ICD code that indicates either major injury or suspicion of abuse and neglect. We will identify major injury through the observation of any medical claim or mortality case including an ICD code associated with injury excluding superficial injuries, injuries related to medical care, and injuries stemming from allergic reactions. ICD codes indicating suspected abuse and neglect are derived from Schnitzer et al. (2011) and Hooft et al. (2013) based on validated methods [69, 70]. Data on early childhood injury outcomes and suspected abuse and neglect will come from South Carolina all-payer hospital discharge records, Medicaid inpatient and outpatient claims, and mortality records. Previous work examining the impact of home visiting programs on abuse and neglect has considered the impact of the program on investigation for child abuse and neglect [71]. We focus on health care encounters and mortality in order to mitigate potential reporting bias introduced because NFP home visiting nurses are mandated reporters of child abuse and neglect. We will consider the impact of the program on investigations of abuse and neglect and confirmed cases of abuse and neglect using data provided by the Department of Social Services in exploratory analyses. In order to account for the possibility that control and treatment groups have different Medicaid eligibility and are therefore differentially likely to appear in Medicaid claims, we will also present a robustness check estimating the impact of NFP on major injuries and suspected abuse and neglect appearing solely in hospital discharge records. A list of ICD-10 codes that will be used to define major injuries are presented in Table 2. ICD-9 codes used to define suspected abuse and neglect are presented in Table 3. We use General Equivalence Mappings from the Centers for Medicare and Medicaid Services (CMS) to convert from ICD-9 to ICD-10 codes, as validated codes for suspected abuse and neglect are published using ICD-9 codes only [72]. For mothers with multiple births, we define the outcome based on having any code indicating major injury or suspicion of abuse and neglect for any child. We will also consider secondary outcomes which capture utilization of emergency care and adherence with preventative guidelines and well-child care (Table 1).

**Table 2:** Injury outcome definition

ICD-10-CM Code	Code description	ICD-10 exclusion codes
S00-S09	Injuries to the head	S00-S00.9 (Superficial injuries of the head)
S10-S19	Injuries to the neck	S10-S10.9 (Superficial injuries of the neck)
S20-S29	Injuries to the thorax	S20-S20.8 (Superficial injuries of the thorax)
S30-S39	Injuries to the abdomen, lower back, lumbar spine, and pelvis	S30-S30.9 (Superficial injuries of the abdomen, lower back, and pelvis)
S40-S49	Injuries to the shoulder and upper arm	S40-S40.9 (Superficial injuries of the shoulder and upper arm)
S50-S59	Injuries to the elbow and forearm	S50-S50.9 (Superficial injuries of the elbow and forearm)
S60-S69	Injuries to the wrist and hand	S60-S60. 9 (Superficial injuries of the wrist and hand)
S70-S79	Injuries to the hip and thigh	S70-S70.9 (Superficial injuries of the hip and thigh)
S80-S89	Injuries to the knee and lower leg	S80-S80.9 (Superficial injuries of the knee and lower leg)
S90-S99	Injuries to the ankle and foot	S90-S90.9 (Superficial injuries of the ankle and foot)
T00-T07	Injuries involving multiple body regions	T00-T00.9 (Superficial injuries involving multiple body regions)
T08-T14	Injuries to unspecified part of trunk, limb, or body region	T09.0 (Superficial injury of the trunk)
T15-T19	Effects of foreign body entering through natural orifice	
T20-T32	Burns and corrosions	
T33-T35	Frostbite	
T36-T50	Poisoning by drugs, medicaments and biological substances	
T51-T65	Toxic effects of substances chiefly nonmedicinal as to source	
T66-T78	Other and unspecified effects of external causes	T78 (Allergies)
T79	Certain early complications of trauma

**Table 3:** Abuse outcome definition

ICD-9-CM Code	Code description
054.1, 098	Genital herpes, Gonococcal infection
995.50^†^, 995.54^†^, 995.55^†^, 995.59^†^	Child physical abuse; Shaken baby syndrome; Other child abuse and neglect, not otherwise specified
262	Other severe malnutrition
362.81	Retinal hemorrhage
521.0	Dental caries
614.9	Pelvic inflammatory disease, unspecified
692.7	Solar radiation dermatitis
800^†^, 805^†^, 807.0^†^, 807.1^†^, 808^†^, 811^†^	Skull vault fracture; Vertebral fracture; Rib Fracture; Pelvic fracture; Scapula fracture
852.0^†^, 852.2^†^, 852.2^†^	Traumatic subarachnoid hemorrhage; Traumatic subdural hemorrhage; Other/unspecified intracranial hemorrhage
860^†^	Traumatic pneumohemothorax
861^†^, 862^†^	Heart or lung injury; intrathoracic injury, not elsewhere classified
863.1^†^, 863.2^†^, 863.3^†^, 863.8^†^	Stomach injury; Small intestine injury; GI injury not elsewhere classified
864^†^	Liver injury
865^†^	Spleen injury
866^†^	Kidney injury
922.4	Contusion of genital organs
941^†^, 942^†^, 945^†^, 946^†^	Burn of head: Burn of trunk; Burn of leg; Burn of multiple sites
952^†^	Spinal cord injury
960–979^†^	Poisoning by drugs/medicinals
994.1^†^	Drowning, non-fatal submersion
E869.4	Second-hand tobacco smoke
E910.2, E910.4, E910.8, E910.9	Swimming accident, Bathtub (near) drowning, Other (near) drowning, Accidental (near) drowning, not otherwise specified
E960.0; E961; E962; E963; E964; E965; E966; E967; E968.0; E968.1; E968.2; E968.3, E968.4, E968.5, E968.6, E968.7, E968.8, E968.9, E980, E985, E988, V60, V71.5, V71.81	Unarmed fight, brawl; Assault by corrosive or caustic substance, except poisoning; Assault by poisoning; Assault by hanging and strangulation; Assault by submersion; Assault by firearms and explosives; Assault by cutting and piercing instrument; Perpetrator of child and adult abuse; Assault by fire; Assault by pushing from a high place; Assault by striking by blunt or thrown object; Assault by hot liquid; Assault by criminal neglect; Assault by transport vehicle; Assault by air gun; Assault by human bite; Assault by other specified means; Assault by unspecified means; Undetermined intent, poisoning; Undetermined intent, firearm; Undetermined intent, other means; Household circumstances; Observation after alleged rape; Observation for abuse/neglect

† Indicates overlap between abuse and injury definitions. Cases that fall under both the definition of injury and abuse will only be counted once towards the overall metric

**Fig. 3:**
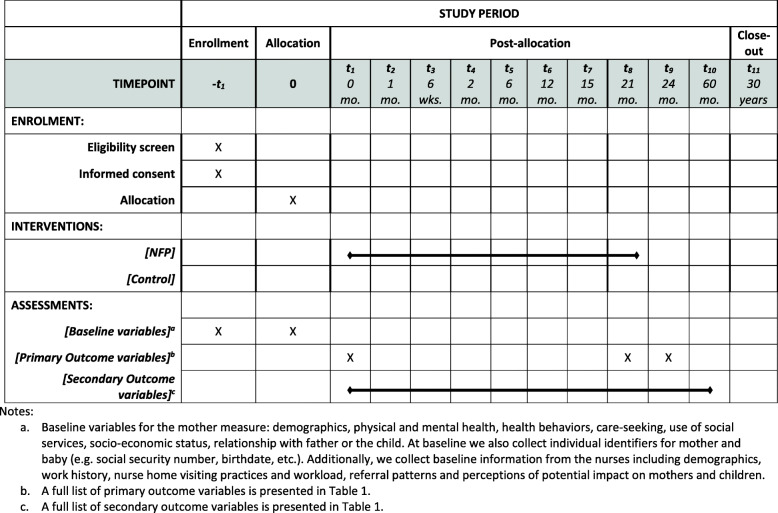
Schedule of enrollment, interventions, and assessments. Notes: (a) Baseline variables for the mother measure: demographics, physical and mental health, health behaviors, care-seeking, use of social services, socioeconomic status, relationship with father or the child. At baseline, we also collect individual identifiers for mother and baby (e.g., social security number, birthdate). Additionally, we collect baseline information from the nurses including demographics, work history, nurse home visiting practices and workload, referral patterns and perceptions of potential impact on mothers and children. (b) A full list of primary outcome variables is presented in Table 1. (c) A full list of secondary outcome variables is presented in Table 1

Third, we will assess the effect of NFP on birth spacing. We define the outcome based on whether a subsequent birth occurs less than 21 months after the birth of the child born from the pregnancy during which the mother was enrolled in the study. Studies examining various pregnancy interval lengths find stronger and more consistent effects on infant health for birth intervals of 6 and 12 months [43]. We selected a birth interval of 21 months, corresponding to a 12-month pregnancy interval, in order to capture the potential implications for infant health, ensure adequate statistical power for detecting effects, and reflect preferences of the vast majority of mothers enrolled in the study who report a desire to space births by at least two years (96.7% of currently enrolled sample). Data for this outcome will come from South Carolina birth certificate records. Secondary outcomes will consider utilization of family planning and contraceptive services. While South Carolina has not expanded Medicaid under the Affordable Care Act, South Carolina has a Medicaid Waiver to provide family planning services for individuals with the same income eligibility criteria as the state’s Medicaid eligibility threshold during pregnancy [73]. We provide a SPIRIT figure as Fig. 3 detailing activities surrounding enrollment, interventions, and assessments.

Our primary study outcomes are not defined identically to outcomes defined in the PFS contract. The outcomes in the PFS contract were defined by the signatories of the contract through the contracting process. We subsequently refined the definitions for the analyses in this protocol paper based on the literature on home visiting and an assessment of which outcomes our study would have sufficient statistical power to detect. Per the PFS contract schedule, the PFS outcomes will also be reported based on a partial sample, whereas the analyses described here will be reported on the full study sample. For these two reasons, we expect that the results of the analysis described in this protocol paper will likely differ from the point estimates we will provide to the PFS contract signatories.

### Subgroup analysis

Similar to previous NFP trials, we will examine differential impacts of the program among a subgroup of mothers whose circumstances at enrollment place them and their children at increased risk of adverse outcomes [22, 74, 75]. This primary subgroup includes mothers who have some indicator of poor mental health, are under 19 years old, or have not completed high school or received a General Education Development certificate by the time of study intake. We define the mental health indicator as either having received mental health treatment in the last year, or having elevated depressive symptoms as measured using the abbreviated Patient Health Questionnaire (PHQ-2), with scores of 3 or greater indicating high likelihood of a major depressive disorder [76]. Our focus on families experiencing greater perceived mental health challenges is consistent with previous subgroup analyses from the Memphis and Denver trials evaluating NFP that have focused on mothers with “low psychological resources” (including poor mental health outcomes). Our classification of mothers under 19 years old as high risk mirrors the criteria used to define the most vulnerable mothers in the trial of NFP conducted in Elmira [62]. We use the completion of high school as an indicator of potential economic mobility, whereas the Elmira trial measures low socioeconomic status using the Hollingshead Four Factor index [77].

We may also consider the program’s differential impact among other secondary subgroups of particular interest to policy-makers or stakeholders. For example, we may consider how program impacts on birth outcomes differ by maternal race, how outcomes on child injury and suspected abuse and neglect differ for a subgroup identified as experiencing substance abuse disorder, or how birth spacing differs for mothers who had expressed a desire to avoid closely spaced pregnancies. We will fully specify planned subgroups as we develop more detailed pre-analysis plans for specific academic manuscripts. When discussing heterogeneity in treatment impacts, we will clearly indicate whether subgroup analyses were planned and documented in a pre-analysis plan or are exploratory. Finally, for any subgroup analyses, we will correct for multiple hypothesis testing as discussed in “Statistical power” section.

### Attrition

Previous evaluations of NFP have been able to track families with in-person surveys over long periods of time with low to moderate attrition rates, with follow-up rates between 62 and 93% depending on the outcome of interest and the length of follow-up [64, 65]. Though our use of administrative data to measure study outcomes mitigates many challenges associated with long-term follow-up, our study faces other potential sources of attrition, in particular due to miscarriage or out-of-state migration. Specifically, we will not observe outcomes for mothers who experience a miscarriage early in pregnancy. Considering the distribution of gestational age at enrollment from our baseline survey and estimates of miscarriage risk by gestational week [78], we estimate that approximately 5% of mothers enrolled into the study will experience a miscarriage. In terms of out-of-state migration, South Carolina experiences a 3% out-migration over the course of 1 year (based on data from the census), and we anticipate that the rate of out-migration may be slightly lower among our sample of low-income first-time mothers. Evidence suggests that moving during the pregnancy and postpartum period often occurs over limited distances (< 10 km) [79]. We may also see attrition due to inability to match mothers to administrative records because of inadequate identifying information. We anticipate that we will be unable to match an estimated 3% of mothers enrolled in the sample for this reason. Finally, we anticipate that some mothers will withdraw their consent to participate in the study, though we anticipate this will account for < 1% of the enrolled sample.

For the purposes of statistical power calculations (“Statistical power” section), we calculate statistical power assuming a lower bound of 7.5% attrition and an upper bound of 15% attrition across both treatment and control groups through miscarriage, migration, imperfect identifiers for matching, or withdrawing consent. We anticipate that our use of administrative data will limit differential attrition across study arms. However, if attrition is higher than expected or differs substantially between treatment arms, we will conduct a bounding exercise to understand the robustness of our estimated treatment effects to possible attrition patterns [80].

### Planned analyses

We will estimate intent-to-treat (ITT) effects as the primary empirical specification. We will also report local average treatment effects (LATE). The ITT estimates capture differences in outcomes between those assigned to the treatment group and those assigned to the control group. The LATE estimates incorporate information on actual program participation, taking advantage of the randomization as an instrument for participation and allowing examination of average characteristics of those participating in the program [81]. We define program participation as receiving at least one visit from a nurse. Intervention group status is used as an instrumental variable for program participation. Consider an outcome, *Y*_*i*_, such as an indicator for an adverse birth outcome. For subject *i*, the estimating equation is:

$$ {Y}_i={\pi}_0+{\pi}_1I{\left(\mathrm{Enrolled}\ \mathrm{in}\ \mathrm{NFP}=1\right)}_i+{\pi}_2{X}_i+{\vartheta}_i $$

where “Enrolled in NFP” means having received at least one completed visit from NFP for service delivery. This model will be estimated using two-stage least squares (2SLS), where the first stage is:

$$ I{\left(\mathrm{Enrolled}\ \mathrm{in}\ \mathrm{NFP}=1\right)}_i={\alpha}_0+{\alpha}_1I{\left(\mathrm{Treatment}=1\right)}_i+{\alpha}_2{X}_i+{\omega}_i $$

where *I*(Treatment = 1)_*i*_ is an indicator variable equal to one if the subject was randomized to the intervention group and zero if the subject was randomized to the control group; *X*_*i*_ is a vector of covariates, specified in more detail below. These covariates should be uncorrelated with the treatment indicator because of the randomization. We include them in the model since they may increase the precision of the estimates. This linear model estimates the local average treatment effect (LATE) of NFP on intervention group members who actually participate in NFP relative to the services consumed by the control group. This estimated effect of NFP is of policy interest because it represents the impact of NFP on those clients who are likely to participate in NFP were the program to expand and offer additional program slots through a lottery. The source of non-compliance that it explicitly captures is that some mothers randomized into the intervention group may never receive NFP services (i.e., the “enrollment rate” is less than 1). According to the enrollment protocol, no mothers in the control group should be enrolled in NFP services. To the extent that some sample members in the control group receive services from similar home visiting programs that may also affect outcomes, this model estimates the effect of NFP relative to the mix of other home visiting programs that the control group receives, rather than relative to no home visiting service at all. We may also consider additional secondary specifications that may be of interest to policy-makers including an estimate of local average treatment effects where we consider two potentially relevant alternative definitions of “treatment:” (1) mothers who are still receiving home visits at the time of their anticipated delivery date as reported on the baseline survey and (2) mothers who receive home visits throughout the entire program period of pregnancy and the child’s first 2 years of life.

Existing literature and previous NFP trials have identified a number of characteristics that may be predictive of the three primary study outcomes. Such characteristics include maternal age, indicators of race and ethnicity, health behaviors (smoking and drinking), maternal socioeconomic status, relationship to father of the child, utilization of health services, and mental health. In our impact models, we will include variables from the baseline survey to measure these and other relevant sample characteristics (including gestational age at enrollment and indicators for implementing agency of NFP).

### Statistical power

We calculate minimum detectable differences reported as the percentage point difference between treatment and control groups we will be able to detect, assuming a significance level (alpha) of 5% and a statistical power level of 80%. Based on current enrollment trends, we assume 96% of study participants randomized into the treatment group will receive NFP services. Furthermore, we assume that none of the control group receives NFP services. We will account for multiple hypothesis testing across three different outcomes and within one subgroup by calculating and reporting false discovery rate (FDR)-adjusted *p* values across all primary outcomes and subgroup analyses (6 hypothesis tests in total) using methods developed by Benjamini and Hochberg [82, 83]. We provide calculations based on a lower-bound and upper-bound estimate as discussed in “Subgroup analysis” section. We report both unadjusted and Bonferroni-adjusted minimum detectable differences for each outcome in Table 4. Because the FDR method improves power over the Bonferroni correction, Bonferroni-adjusted minimum detectable differences should be interpreted as upper bounds. Our estimates of control group means for our three primary outcomes are determined as follows. *Adverse Birth Outcomes*: We use vital records data from South Carolina to estimate that approximately 24.3% of study participants will have an adverse birth outcome in our control group. *Birth Spacing*: Assumptions about the control group mean for the birth spacing outcome come from recently published evidence on the incidence of shortly spaced births in South Carolina [84]. *Early childhood injuries or suspected abuse and neglect*: The assumptions about the control group mean for the early childhood injury outcome are estimated based on analysis from a sample of hospital discharge data from South Carolina. We anticipate that 18% of families in our sample will experience injury, abuse, or neglect.

**Table 4:** Power calculations

Primary outcomes	Assumptions regarding control group mean	Full sample (unadjusted)		Full sample (Bonferroni)		Vulnerable subgroup (unadjusted)		Vulnerable subgroup (Bonferroni)	
		*Low attrition*	*High attrition*	*Low attrition*	*High attrition*	*Low attrition*	*High attrition*	*Low attrition*	*High attrition*
Adverse birth outcome	24%	− 3.5 pp. (14%)	− 3.6 pp. (15%)	− 4.3 pp. (18%)	− 4.4 pp. (18%)	− 5.2 pp. (21%)	− 5.4 pp. (22%)	− 6.3 pp. (26%)	− 6.6 pp. (27%)
Birth spacing	13%	− 2.7 pp. (20%)	− 2.8 pp. (21%)	− 3.3 pp. (25%)	− 3.4 pp. (26%)	− 3.9 pp. (30%)	− 4.1 pp. (31%)	− 4.8 pp. (36%)	− 5.0 pp. (38%)
Acute injury, abuse or neglect	18%	− 3.1 pp. (17%)	− 3.2 pp. (18%)	− 3.8 pp. (21%)	− 3.9 pp. (22%)	− 4.6 pp. (26%)	− 4.7 pp. (26%)	− 5.6 pp. (31%)	− 5.8 pp. (32%)

Note(s):

Power calculations show the minimum detectable effect between treatment and control in percentage points (pp) with percentages in parentheses. Percentages are rounded to the nearest whole percent. We assume a significance level (alpha) of 5% and a statistical power level of 80%, and that 96% of study participants randomized into the treatment group will receive NFP services, based on current enrollment trends. Additionally, we assume that none of the control group receives NFP services. Low attrition calculations assume 7.5% attrition, while High attrition estimates assume 15% attrition across both treatment and control groups through miscarriages, migration, imperfect identifier for matching, or withdrawing consent

### Study registration, pre-analysis plan, and reporting of results

Planned analyses will be pre-specified in a publicly archived analysis plan prior to un-blinding the data for analysis. With the exception of contractually required interim reporting on outcomes that are part of the PFS contract (scheduled for April 2021), data will be unblinded sequentially as outcomes can be observed for the full sample (Fig. 2). We may report on primary outcomes in separate manuscripts, for example reporting on birth outcomes prior to reporting on child outcomes which occur later in time. We will create and publish pre-analysis plans for separate planned manuscripts (including the analysis of pre-specified secondary outcomes and of additional domains and longer-term outcomes such as those in Table 1) before conducting analysis on outcomes using treatment assignment. This practice strengthens the integrity of the inferences drawn, facilitating multiple-inference adjustment and guarding against external pressures. We have registered the trial on the American Economics Association’s registry of social science RCTs and on Clinicaltrials.gov. Following the conclusion of the publication of primary study outcomes, we will make a de-identified public use dataset and replication code available to the maximum extent that is legally permissible under the terms of our data use agreements. As this study is not a traditional clinical trial, the study does not have a Data Monitoring Committee.

### Trial status

The study concluded enrollment earlier than expected on March 17, 2020, because of safety concerns related to the COVID pandemic. The originally planned study population was 6000 mothers total (4000 in the treatment group and 2000 in the control group) to be enrolled over 4 years (April 2016–2020), with NFP services delivered through the children’s second birthday (2016–2022). At the conclusion of enrollment, 5655 were enrolled in the study. This protocol was first drafted on January 30, 2020, and revised on October 21, 2020, in response to reviewer comments.

## Discussion

In this protocol, we outline major objectives of NFP’s home visiting services and define scientific primary and secondary outcomes related to maternal and child health that can be observed during the prenatal period through the first 24 months of life. These outcomes relate to the central goals of home visiting programs to improve pregnancy and birth outcomes, to reduce child injuries and incidence of abuse and neglect, and to help mothers alter their life course by achieving their desired birth spacing.

Our evaluation will assess the impacts that might be expected from NFP operating at scale. Previous NFP studies highlighted the impacts of the program on important subgroups, such as unmarried or teenage mothers [22, 62, 75] and “low-resourced” mothers [85], so we will also examine the effect in a subgroup of mothers who may experience particular challenges [25]. Our analysis will be powered to detect whether program impacts across the three key domains defined in this protocol differ for this key population.

The opportunity to apply the scientific rigor of a randomized controlled trial to assess the impact of NFP at scale was made possible by a federal Medicaid waiver and a collaboration between SCDHHS and philanthropic organizations on a Pay for Success contract. This will be the largest randomized trial linked to a Pay for Success project to date, as well as among the largest trials of an early life intervention among low-income mothers in the USA. While the PFS structure enabled this randomized controlled trial, it necessitates a different framework for reporting results than is typical for purely academic studies. First, as independent evaluators to the PFS contract, we are required to report on the PFS outcomes for an incomplete sample prior to the observation of these outcomes for the entire sample. This means that estimates of outcomes used for payments may differ from final scientific output.

Second, the primary scientific outcomes we have chosen differ from the PFS outcomes in several ways. While the PFS contract focuses on the costly outcome of preterm birth, we define a composite of adverse birth outcomes that includes preterm birth, low birth weight, or small for gestational age in order to increase power and to detect a broader set of potential pathways to impact around improving birth outcomes. Furthermore, while the PFS birth spacing outcome focuses on subsequent births that occur less than 24 months from the first birth, we pre-specify a scientific outcome of births spaced less than 21 months based on evidence surrounding birth intervals more closely linked to adverse infant health outcomes [43] and on the desired fertility intentions of mothers reported at the time of enrollment in our sample. Finally, while the third PFS outcome focuses on child injuries that generate a hospital or Emergency Department visit, we specify a composite outcome that combines either major child injury (excluding superficial injuries) or suspected abuse and neglect based on recent literature documenting child maltreatment in administrative claims records [69, 70]. In all cases, we will report on the outcome defined for the PFS project among our study’s secondary outcomes.

Relying on existing administrative data, rather than collecting new primary data, offers several advantages. First, utilizing administrative data reduces concerns about likely differential attrition between treatment and control group members in the years following study enrollment. Second, since the data are already being collected for a purpose separate from the study, the chance of data quality differences between the treatment and control groups is minimized. Finally, administrative data, in general, are less likely than primary data to suffer from recall bias or the impulse to please data collectors, and can often provide more granularity than surveys. Using administrative data to measure our outcomes also enables us to track outcomes robustly over a long time horizon in a large sample. One drawback of relying exclusively on administrative data, however, is that it limits our analysis to the outcomes that are already in existing data sets, which may not include some important outcomes that could be affected by NFP. For example, we will not be able to measure cognitive development in early childhood, as South Carolina does not universally conduct screening before children enter school. In addition, we will not directly observe psychosocial outcomes that are directly related to NFP’s focus on the quality of parenting.

A final potential challenge to using administrative data for measuring outcomes is that we will not be able to observe outcomes related to outpatient care delivered by commercial payers or accessed at safety net providers. Furthermore, participation in the NFP program may impact whether families are eligible for Medicaid, either directly through helping families with application and renewal processes or indirectly through income eligibility. This could mean that the sample of mothers whose outcomes will be observed in Medicaid claims data could differ across study arms. We will account for this possibility by comparing enrollment rates in Medicaid across treatment arms, by considering alternative specifications that focus only on discharge records, which can be observed for all study participants, and by conducting bounding exercises [80] to gauge the robustness of estimated treatment effects if we see differential enrollment rates across arms.

Beyond the pre-specified primary and secondary health-related outcomes that we will observe over the period of program implementation discussed in this protocol, the randomized controlled trial provides an opportunity to measure the effect of NFP across a much wider set of outcomes and longer time horizon. In several recent studies, early childhood programs were found to have limited effects in the short to medium run, but notable effects in the very long run [86–88]. Having obtained consent to follow the study participants and their children for up to 30 years, in future work we will be able to provide a more comprehensive assessment of NFP’s impact on intergenerational poverty, health, and well-being. We will utilize an existing administrative data infrastructure to assess a wide range of outcomes, including those beyond health and income, such as connection to social services, criminal justice involvement, school performance, and economic well-being.

## Conclusion

Evidence from this trial on the impact of home visiting services delivered at scale as part of a Medicaid benefit can provide policy-makers and stakeholders with crucial information about the effectiveness of the Nurse-Family Partnership program in improving health and well-being for low-income mothers and children. In particular, this study evaluates a program that was previously studied at a relatively small scale including effects seen within a range of distinct subgroups. This evaluation will provide policy-makers with estimates of the NFP’s program when implemented at scale by well-established local service providers. The evaluation also provides an opportunity to measure the comprehensive effects of a health program that operates outside the traditional health care delivery infrastructure and has the potential to impact both health and non-health outcomes.

## Supplementary information

**Additional file 1.** 2013 SPIRIT Checklist. Standard Protocol Items Recommended for International Trials.

**Additional file 2.** PFS Outcomes and Payment Thresholds. Definition of PFS outcomes and thresholds for “success payments”.

**Additional file 3.** Consent Form. Consent form for Participants enrolled in NFP Trial.

**Additional file 4.** Resources for New Moms and Babies: Greenville County. Example list of resources available to low-income mothers. List provided to both control and treatment group enrollees in the study.

**Additional file 5.** Baseline Survey. Baseline survey administered to all participants enrolled in the trial after informed consent and before randomization.

**Additional file 6.** Home Visitor Survey. Survey administered to all nurse home visitors before enrollment for the trial is complete.

## Data Availability

Following the conclusion of the trial and publication of results regarding primary outcomes, we will make a de-identified public use dataset and replication code available to the maximum extent that is legally permissible under the terms of our data use agreements. The research team has prior experience producing data and documentation that are accessible to outside researchers.

## Abbreviations

2SLS: Two-stage least squares
AAP: American Academy of Pediatrics
AAPD: American Academy of Pediatric Dentistry
CAPI: Computer-assisted personal interview
CDC: Centers for Disease Control and Prevention
DHEC: Department of Health and Environmental Control
FDR: False discovery rate
ICD: International Classification of Diseases
ITT: Intent-to-treat
J-PAL: Abdul Latif Jameel Poverty Action Lab
LATE: Local average treatment effect
LBW: Low birth weight
MIECHV: Maternal, Infant, and Early Childhood Home Visiting
NBER: National Bureau of Economic Research
NFP: Nurse-Family Partnership
PFS: Pay for Success
PHQ-2: Patient Health Questionnaire
SCDHHS: South Carolina Department of Health and Human Services

## References

[1] National Academies of Sciences E (2019). A roadmap to reducing child poverty.

[2] Ratcliffe C, McKernan S-M. Childhood poverty persistence: facts and consequences: Urban Institute; 2016. https://www.urban.org/research/publication/childhood-poverty-persistence-facts-and-consequences. Accessed 21 Dec 2019.

[3] Almond D, Currie J, Duque V (2018). Childhood circumstances and adult outcomes: act II. J Econ Lit.

[4] Almond D, Currie J (2011). Killing me softly: the fetal origins hypothesis. J Econ Perspect J Am Econ Assoc.

[5] Aizer A, Currie J (2014). The intergenerational transmission of inequality: maternal disadvantage and health at birth. Science.

[6] Jones NL, Gilman SE, Cheng TL, Drury SS, Hill CV, Geronimus AT (2019). Life course approaches to the causes of health disparities. Am J Public Health.

[7] Importance of Social Determinants of Health and Cultural Awareness in the Delivery of Reproductive Health Care - ACOG. https://www.acog.org/Clinical-Guidance-and-Publications/Committee-Opinions/Committee-on-Health-Care-for-Underserved-Women/Importance-of-Social-Determinants-of-Health-and-Cultural-Awareness-in-the-Delivery-of-Reproductive. Accessed 21 Dec 2019.

[8] Pediatrics C on C (2016). Poverty and Child Health in the United States. Pediatrics.

[9] Garg A, Boynton-Jarrett R, Dworkin PH (2016). Avoiding the unintended consequences of screening for social determinants of health. JAMA.

[10] Ickovics JR, Kershaw TS, Westdahl C (2007). Group prenatal care and perinatal outcomes: a randomized controlled trial. Obstet Gynecol.

[11] Mazzoni SE, Carter EB (2017). Group prenatal care. Am J Obstet Gynecol.

[12] Hale N, Picklesimer AH, Billings DL, Covington-Kolb S (2014). The impact of centering pregnancy group prenatal care on postpartum family planning. Am J Obstet Gynecol.

[13] Bohren MA, Hofmeyr GJ, Sakala C, Fukuzawa RK, Cuthbert A (2017). Continuous support for women during childbirth. Cochrane Database Syst Rev.

[14] McGrath SK, Kennell JH (2008). A randomized controlled trial of continuous labor support for middle-class couples: effect on cesarean delivery rates. Birth Berkeley Calif.

[15] Everson CL, Cheyney M, Bovbjerg ML (2018). Outcomes of care for 1,892 doula-supported adolescent births in the United States: the DONA international data project, 2000 to 2013. J Perinat Educ.

[16] Kozhimannil KB, Vogelsang CA, Hardeman RR, Prasad S (2016). Disrupting the pathways of social determinants of health: doula support during pregnancy and childbirth. J Am Board Fam Med.

[17] Kozhimannil KB, Hardeman RR, Attanasio LB, Blauer-Peterson C, O’Brien M (2013). Doula care, birth outcomes, and costs among Medicaid beneficiaries. Am J Public Health.

[18] Shonkoff JP, Garner AS, Committee on Psychosocial Aspects of Child and Family Health, Committee on Early Childhood, Adoption, and Dependent Care, Section on Developmental and Behavioral Pediatrics (2012). The lifelong effects of early childhood adversity and toxic stress. Pediatrics.

[19] Shonkoff JP, Fisher PA (2013). Rethinking evidence-based practice and two-generation programs to create the future of early childhood policy. Dev Psychopathol.

[20] Olds DL, Robinson J, Pettitt L (2004). Effects of home visits by paraprofessionals and by nurses: age 4 follow-up results of a randomized trial. Pediatrics.

[21] Olds DL, Henderson CR, Tatelbaum R, Chamberlin R (1988). Improving the life-course development of socially disadvantaged mothers: a randomized trial of nurse home visitation. Am J Public Health.

[22] Kitzman H, Olds DL, Henderson CR (1997). Effect of prenatal and infancy home visitation by nurses on pregnancy outcomes, childhood injuries, and repeated childbearing: a randomized controlled trial. JAMA.

[23] Haskins R, Margolis G. Show me the evidence: Obama’s fight for rigor and results in social policy: Brookings Institution Press; 2015. www.jstor.org/stable/10.7864/j.ctt7zsvr9. Accessed 18 Nov 2019.

[24] Robling M, Bekkers M-J, Bell K (2016). Effectiveness of a nurse-led intensive home-visitation programme for first-time teenage mothers (building blocks): a pragmatic randomised controlled trial. Lancet.

[25] Publications | Home Visiting Evidence of Effectiveness. https://homvee.acf.hhs.gov/publications/HomVEE-Summary. Accessed 18 Nov 2019.

[26] Dodge KA, Goodman WB, Murphy R, O’Donnell K, Sato J (2013). Toward population impact from home visiting. Zero Three.

[27] Iovan S, Lantz PM, Shapiro S (2018). “Pay for success” projects: financing interventions that address social determinants of health in 20 countries. Am J Public Health.

[28] Duffee JH, Mendelsohn AL, Kuo AA, et al. Early childhood home visiting. Pediatrics. 2017;140(3) 10.1542/peds.2017-2150. 10.1542/peds.2017-2150 28847981

[29] Hodgkinson S, Godoy L, Beers LS, Lewin A. Improving mental health access for low-income children and families in the primary care setting. Pediatrics. 2017;139(1) 10.1542/peds.2015-1175. 10.1542/peds.2015-1175 PMC5192088 27965378

[30] Daw JR, Hatfield LA, Swartz K, Sommers BD (2017). Women in the United States experience high rates of coverage ‘churn’ in months before and after childbirth. Health Aff (Millwood).

[31] Wallace D, Chamberlain A, Pfeiffer D. The relationship between foreclosures and intimate partner violence during the U.S. housing crisis. J Interpers Violence. 2018:0886260518818431. 10.1177/0886260518818431. 10.1177/0886260518818431 30556475

[32] Housing And Health: An Overview Of The Literature | Health Affairs. https://www.healthaffairs.org/do/10.1377/hpb20180313.396577/full/. Accessed 18 May 2019.

[33] Farrell CA, Fleegler EW, Monuteaux MC, Wilson CR, Christian CW, Lee LK. Community poverty and child abuse fatalities in the United States. Pediatrics. 2017;139(5) 10.1542/peds.2016-1616. 10.1542/peds.2016-1616 28557719

[34] From Medical Home to Health Neighborhood: Transforming the medical home into a community-based health neighborhood - The Journal of Pediatrics. https://www.jpeds.com/article/S0022-3476(12)00020-0/abstract. Accessed 19 May 2019. 10.1016/j.jpeds.2012.01.001 22424405

[35] Lu MC (2018). Reducing maternal mortality in the United States. JAMA.

[36] Martin JA, Hamilton BE, Osterman MJK (2019). Births in the United States, 2018. NCHS Data Brief.

[37] Pregnancy-Related Deaths | CDC. https://www.cdc.gov/reproductivehealth/maternalinfanthealth/pregnancy-relatedmortality.htm. Published February 26, 2019. Accessed 15 Nov 2019.

[38] Howell EA, Brown H, Brumley J (2018). Reduction of peripartum racial and ethnic disparities: a conceptual framework and maternal safety consensus bundle. Obstet Gynecol.

[39] Burris HH, Hacker MR (2017). Birth outcome racial disparities: a result of intersecting social and environmental factors. Semin Perinatol.

[40] Kozhimannil KB, Hung P, Henning-Smith C, Casey MM, Prasad S (2018). Association between loss of hospital-based obstetric services and birth outcomes in rural counties in the United States. JAMA.

[41] Ely DM, Driscoll AK, Matthews TJ (2017). Infant mortality rates in rural and urban areas in the United States, 2014. NCHS Data Brief.

[42] Buckles K, Guldi ME, Schmidt L. Fertility trends in the United States, 1980-2017: the role of unintended births: National Bureau of Economic Research; 2019. 10.3386/w25521.

[43] Ahrens KA, Nelson H, Stidd RL, Moskosky S, Hutcheon JA (2019). Short interpregnancy intervals and adverse perinatal outcomes in high-resource settings: an updated systematic review. Paediatr Perinat Epidemiol.

[44] Barber JS, East PL (2011). Children’s experiences after the unintended birth of a sibling. Demography.

[45] Bailey MJ, Guldi M, Hershbein BJ (2013). Recent evidence on the broad benefits of reproductive health policy. J Policy Anal Manag J Assoc Public Policy Anal Manag.

[46] Bailey MJ, Malkova O, McLaren ZM. Does access to family planning increase children’s opportunities? Evidence from the War on Poverty and the Early Years of Title X. J Hum Resour. 2018:1216-8401R1. 10.3368/jhr.55.1.1216-8401R1. 10.3368/jhr.54.4.1216-8401R1 PMC6876122 31768076

[47] Shah AK, Mullainathan S, Shafir E (2012). Some consequences of having too little. Science..

[48] Schilbach F, Schofield H, Mullainathan S (2016). The psychological lives of the poor. Am Econ Rev.

[49] Mani A, Mullainathan S, Shafir E, Zhao J (2013). Poverty impedes cognitive function. Science.

[50] Bertrand M, Mullainathan S, Shafir E (2004). A behavioral-economics view of poverty. Am Econ Rev.

[51] Mersky JP, Berger LM, Reynolds AJ, Gromoske AN (2009). Risk factors for child and adolescent maltreatment: a longitudinal investigation of a cohort of inner-city youth. Child Maltreat.

[52] Lane WG, Dubowitz H, Langenberg P, Dischinger P (2012). Epidemiology of abusive abdominal trauma hospitalizations in United States children. Child Abuse Negl.

[53] Nemeroff CB (2016). Paradise lost: the neurobiological and clinical consequences of child abuse and neglect. Neuron.

[54] Felitti VJ (2009). Adverse childhood experiences and adult health. Acad Pediatr.

[55] Doyle JJ, Aizer A (2018). Economics of child protection: maltreatment, foster care, and intimate partner violence. Annu Rev Econ.

[56] Karb RA, Subramanian SV, Fleegler EW (2016). County poverty concentration and disparities in unintentional injury deaths: a fourteen-year analysis of 1.6 million U.S. fatalities. PLoS One.

[57] Reading R, Langford IH, Haynes R, Lovett A (1999). Accidents to preschool children: comparing family and neighbourhood risk factors. Soc Sci Med.

[58] Singh GK, Yu SM (1996). US childhood mortality, 1950 through 1993: trends and socioeconomic differentials. Am J Public Health Wash.

[59] Nurse-Family Partnership; Nurses and mothers: Transformational relationship creating 2-Gen change. https://www.nursefamilypartnership.org/wp-content/uploads/2018/06/Nurses-Mothers.pdf. Accessed 1 Dec 2020.

[60] The maternal, infant, and early childhood home visiting program: partnering with parents to help children succeed. https://mchb.hrsa.gov/sites/default/files/mchb/MaternalChildHealthInitiatives/HomeVisiting/pdf/programbrief.pdf. Accessed 1 Dec 2020.

[61] The maternal, infant, and early childhood home visiting program. https://mchb.hrsa.gov/sites/default/files/mchb/MaternalChildHealthInitiatives/HomeVisiting/pdf/programbrief.pdf. Accessed 1 Dec 2020.

[62] Olds DL, Henderson CR, Tatelbaum R, Chamberlin R (1986). Improving the delivery of prenatal care and outcomes of pregnancy: a randomized trial of nurse home visitation. Pediatrics.

[63] Mother and Infant Home Visiting Program Evaluation (MIHOPE), 2011-2019. Office of Planning, Research & Evaluation | ACF. https://www.acf.hhs.gov/opre/research/project/maternal-infant-and-early-childhood-home-visiting-evaluation-mihope. Accessed 9 Jan 2020.

[64] Olds DL, Eckenrode J, Henderson CR (1997). Long-term effects of home visitation on maternal life course and child abuse and neglect: fifteen-year follow-up of a randomized trial. JAMA.

[65] Olds DL, Kitzman H, Cole R (2004). Effects of nurse home-visiting on maternal life course and child development: age 6 follow-up results of a randomized trial. Pediatrics.

[66] Talge NM, Mudd LM, Sikorskii A, Basso O (2014). United States birth weight reference corrected for implausible gestational age estimates. Pediatrics.

[67] Ferreira-González I, Permanyer-Miralda G, Busse JW (2007). Methodologic discussions for using and interpreting composite endpoints are limited, but still identify major concerns. J Clin Epidemiol.

[68] Wallace ME, Crear-Perry J, Mehta P, Theall KP. Homicide during pregnancy and the postpartum period in Louisiana, 2016-2017. JAMA Pediatr. 2020; 10.1001/jamapediatrics.2019.5853. 10.1001/jamapediatrics.2019.5853 PMC7042931 32011644

[69] Schnitzer PG, Slusher PL, Kruse RL, Tarleton MM (2011). Identification of ICD codes suggestive of child maltreatment. Child Abuse Negl.

[70] Hooft A, Ronda J, Schaeffer P, Asnes AG, Leventhal JM (2013). Identification of physical abuse cases in hospitalized children: accuracy of International Classification of Diseases codes. J Pediatr.

[71] Dodge KA, Goodman WB, Bai Y, O’Donnell K, Murphy RA (2019). Effect of a community agency–administered nurse home visitation program on program use and maternal and infant health outcomes: a randomized clinical trial. JAMA Netw Open.

[72] ICD-10 CM and GEMs | CMS. 2018. https://www.cms.gov/Medicare/Coding/ICD10/2018-ICD-10-CM-and-GEMs. Accessed 1 Dec 2020.

[73] Kearney MS, Levine PB (2009). Subsidized contraception, fertility, and sexual behavior. Rev Econ Stat.

[74] Olds DL, Henderson CR, Chamberlin R, Tatelbaum R (1986). Preventing child abuse and neglect: a randomized trial of nurse home visitation. Pediatrics.

[75] Olds DL, Robinson J, O’Brien R (2002). Home visiting by paraprofessionals and by nurses: a randomized, controlled trial. Pediatrics.

[76] Kroenke L, Spitzer BW, Williams BW (2003). The patient health Questionnaire-2: validity of a two-item depression screener. Med Care.

[77] Gottfried AW (1985). Measures of socioeconomic status in child development research: data and recommendations. Merrill-Palmer Q.

[78] Ammon Avalos L, Galindo C, Li D-K (2012). A systematic review to calculate background miscarriage rates using life table analysis. Birt Defects Res A Clin Mol Teratol.

[79] Bell ML, Belanger K (2012). Review of research on residential mobility during pregnancy: consequences for assessment of prenatal environmental exposures. J Expo Sci Environ Epidemiol.

[80] Manski CF (1990). Nonparametric bounds on treatment effects. Am Econ Rev.

[81] Angrist JD, Pischke J-S (2010). The credibility revolution in empirical economics: how better research design is taking the con out of econometrics. J Econ Perspect.

[82] Benjamini Y, Hochberg Y (1995). Controlling the false discovery rate: a practical and powerful approach to multiple testing. J R Stat Soc Ser B Methodol.

[83] Fink G, McConnell M, Vollmer S (2014). Testing for heterogeneous treatment effects in experimental data: false discovery risks and correction procedures. J Dev Eff.

[84] Steenland MW, Pace LE, Sinaiko AD, Cohen JL (2019). Association between South Carolina Medicaid’s change in payment for immediate postpartum long-acting reversible contraception and birth intervals. JAMA.

[85] Kitzman H, Olds DL, Cole R (2010). Enduring effects of prenatal and infancy home visiting by nurses on children: Age-12 follow-up of a randomized trial. Arch Pediatr Adolesc Med.

[86] Chetty R, Friedman JN, Hilger N, Saez E, Schanzenbach DW, Yagan D. How does your kindergarten classroom affect your earnings? Evidence from Project Star. Q J Econ. 2011; 10.1093/qje/qjr041. 10.1093/qje/qjr041 22256342

[87] Cabot P (1940). A long-term study of children: the Cambridge-Somerville youth study. Child Dev.

[88] Mccord J, Farrington DP, Sayre-Mccord G (2007). Crime and family: selected essays of Joan Mccord.

