# Supplementary material for: Protocol for a randomized controlled trial evaluating the impact of the Nurse-Family Partnership’s home visiting program in South Carolina on maternal and child health outcomes
Source: Trials. 2020 Dec 4;21:997. doi: 10.1186/s13063-020-04916-9 (PMC7716290; doi:10.1186/s13063-020-04916-9)
Supplement: Supplementary file 2 [file 13063_2020_4916_MOESM2_ESM.docx]

Additional File 2

PFS Outcomes and Payment Thresholds

PFS Outcome / Measure	Payment Threshold	Measurement source
Preterm birth	13.5% reduction in preterm births	Birth certificates from vital records
Child Injury	23.4% reduction in hospitalizations for acute child injury in first two years of life	Inpatient and ED hospital encounters from all-payer records
Healthy birth interval	18% reduction in subsequent births within twenty-four months	Birth certificates from vital records
Share of Enrollees from LIZCs	65% of mothers from low-income zip codes at time of enrollment	Baseline survey
