# Supplementary material for: Protocol for a randomized controlled trial evaluating the impact of the Nurse-Family Partnership’s home visiting program in South Carolina on maternal and child health outcomes
Source: Trials. 2020 Dec 4;21:997. doi: 10.1186/s13063-020-04916-9 (PMC7716290; doi:10.1186/s13063-020-04916-9)
Supplement: Supplementary file 3 [file 13063_2020_4916_MOESM3_ESM.docx]

Additional File 3

Participant ID:_________________

Protocol Title: Randomized Evaluation of Nurse Family Partnership in South Carolina
Principal Investigator: Margaret McConnell, PhD
Description of Study Population: First-time mothers
Version Date: 12-18-2018

About this consent form
Please read this form carefully. This form provides important information about participating in research. You have the right to take your time in making decisions about participating in this research. You may discuss your decision with your family, your friends and/or your doctor. If you have any questions about the research or any portion of this form, please ask us. If you decide to participate in this research you will be asked to provide an electronic signature. A copy of the information in this form will be provided to you for your record.

Participation is voluntary
You are invited to take part in this research because you are pregnant with your first child and may be eligible to participate in the Nurse Family Partnership (NFP) home visiting program. It is your choice whether or not to participate. If you choose to participate, you may change your mind and leave the study at any time. Refusal to participate or stopping your participation will involve no penalty or loss of benefits to which you are otherwise entitled.

What you should know about a research study
- Someone will explain this research study to you.
- A research study is something you volunteer for.
- Whether or not you take part is up to you.
- You can choose not to take part in the research study.
- You can agree to take part now and later change your mind.
- Whatever you decide it will not be held against you.
- Feel free to ask all the questions you want before you decide.

What is the purpose of this research?
The purpose of this research is to learn about the effects of the Nurse Family Partnership (NFP) home visiting program on your health and wellness and the health and wellness of your children

How many people will take part in this research?
About 6,000 mothers and their children in South Carolina will take part in this research.

How long will I take part in this research?
We will ask you to complete a 30 minute interview today, or at another convenient time. Using the information you provide during the interview, we will also gather historical and future administrative records about the health and wellbeing of you and your children. We will collect these administrative records for up to 30 years after you enroll, but this data collection will not require you or your children's direct involvement or take up any of your time. We would seek your permission again if we would like to ask you any additional questions that would take up any more of your time in the future.

Participant ID:________________

What can I expect if I take part in this research?

If you choose to take part in this research, we will ask you to complete a 30 minute interview today or at another convenient time. During the interview, we will collect identifiable information from you (such as your name and health plan number). We will talk to you about you and about other members of your household. We will ask about your health, feelings, and use of social services. We will ask you to tell us how we can reach you in the future. We may ask you for the names of people who will know how to reach you. The interview will be audio recorded for quality assurance purposes. This means that about 5 minutes of this interview will be chosen at random to be recorded so that the study team may listen to these recordings to ensure the quality of data collection. The audio recordings will be stored securely and destroyed 7 years after the completion of the study.

After you complete the interview, a computer will randomly decide whether you will be offered a spot in the Nurse Family Partnership (NFP) home visiting program. Two-thirds of mothers in the study will be offered a spot in the NFP program. The other one-third will not be offered a spot in the NFP program, however they will receive information about other services available in the community. You will have the same chance to get the NFP program as everyone else. Picking the mothers who will be offered NFP is done randomly, like tossing a coin. Even if you are not offered a spot in the NFP program, we will use the information you provide during the interview to access historical and future administrative records that relate to the health and wellbeing of you and your children for the study. This may include data on how often you receive home-visiting services, the birth of your children, health care use (such as how many times you visit a health facility), government records, interaction with government services (such as cash assistance, food stamps, or child welfare), credit reports and criminal records, or records from your children's school after they start going to school.

All of the information listed above would be collected about you and your children as part of normal public program administration, regardless of whether you take part in the study. Taking part in the study means that you allow researchers to use this existing information for research purposes.

If you are randomized to receive services

If you are randomized to receive services, you will be offered a spot in the NFP program. If you choose to participate in the NFP program, you will be assigned a nurse who will be available to visit you in your home throughout your pregnancy and until your baby is two years old. During this time, your nurse can visit you in your home about every week or two. You and your nurse will decide the exact schedule.

If you are not randomized to receive services

If you are not randomized to receive services, you will not be offered a spot in the NFP program. You are however eligible for other community services. Your information will still be used in the study and your participation in the study may improve future programs and services for first-time mothers and their children.

What are the risks and possible discomforts?

There is only minimal risk in taking part in this research. There is always a small risk when sharing your Protected Health Information (PHI). However, the researchers are following strict rules to keep this information private. This risk applies to all study members, both those who are offered the Nurse Family Partnership services and those who are not. No reports will include your name or other identifying information.

Participant ID:________________

There is one exception to confidentiality we need to make you aware of. The nurses that are part of our study team must comply with state laws that require them to report to authorities if they have reason to believe a child is being abused or neglected. It is our ethical responsibility to report situations of child abuse, child neglect, or any life-threatening situation to appropriate authorities. However, we are not seeking this type of information in our study, nor will you be asked questions about these issues.

Are there any benefits from being in this research study?
Regardless of whether you are randomly assigned to receive services or not, you may benefit from the list of local resources we provide to you. If you are randomized to receive services, you may also benefit from the access to additional information and support through NFP, which may improve your and your children's outcomes.

What are my alternatives to participating in this research?
The alternative to participating in this research study is not to participate. If you choose not to participate, we will not enter your name for a chance to receive NFP services and we will not collect any data on you or your children.

Can I still get medical care if I choose not to participate in this research?
Yes, you may still get medical care if you choose not to participate in this study. Your decision will not change the medical care you receive now or in the future. Taking part in this research is your choice. If you decide to take part in this study, you may leave/stop the study at any time. There will be no penalty to you and your medical care will not be affected. If you would like to stop participating in this research you should let us know.

Will I be compensated for participating in this research?
To compensate you for the time it takes you to complete the survey, you will receive a $25 gift card.

What will I have to pay for if I participate in this research?
Nothing. It will not cost you anything to participate in this research.

What happens if I am injured as a result of participating in this research study?
If physical injury resulting from participation in this research should occur, although Harvard's policy is not to provide compensation, medical treatment will be available including first aid, emergency treatment and follow-up care as needed, and your insurance carrier may be billed for the cost of such treatment. In making such medical treatment available, or providing it, the persons conducting this research project are not admitting that your injury was their fault.

Can my taking part in the research end early?
Yes. You may decide to stop participating in the research at any time without it being held against you.

If I take part in this research, what happens to the information you collect?
Data collected, including your identifiable information, may be seen by researchers and the staff involved in this research study and the Harvard T.H. Chan School of Public Health Institutional Review Board (IRB) that oversees the research. To help maintain the privacy of your study records, you will be assigned a subject number. All of your information from the study will be kept with only your subject number. Your information from the study will not be kept with things like your name and address, or social security number that could identify you. Your study documents will be stored in a locked file cabinet or a

secure electronic storage system. Findings from this study may be published in scientific journals, meetings or public data sets. However, only average results for a group of study participants will be reported; no one will be able to identify you or your children in any of these publications.

If I have any questions, concerns or complaints about this research study, who can I talk to?

The Principal Investigator of this study: Margaret McConnell, PhD, can be reached via email (mmcconne@hsph.harvard.edu) or via mail (677 Huntington Avenue, Building 1, Room 1217, Boston, Massachusetts 02115).

- If you have questions, concerns, or complaints,
- If you would like to talk to the research team,
- If you think the research has hurt you, or
- If you wish to withdraw from the study.

This research has been reviewed by a Harvard Longwood Medical Area Institutional Review Board (Harvard T.H. Chan School of Public Health). If you wish to speak with someone from the IRB, please contact the Office of Human Research Administration (OHRA) at 617-432-2157 (or toll-free at 1-866-606-0573) or 90 Smith Street, Boston, Massachusetts 02120 for any of the following:

- If your questions, concerns, or complaints are not being answered by the research team,
- If you cannot reach the research team,
- If you want to talk to someone besides the research team,
- If you have questions about your rights as a research participant, or
- If you want to get information or provide input about this research.

Authorization to Use and/or Share Your Protected Health Information (PHI)

Federal law requires Harvard University to protect the privacy of health information that identifies you. This information is called Protected Health Information. In the rest of this section, we refer to this simply as "health information."

If you decide to take part in this research study, your health information may be used within Harvard University and may be shared with other research staff outside of Harvard University.

We have marked with a ☒ how we plan to use and share your health information. If a box is not checked ☐, it means that type of use or sharing is not planned for this research study.

- **Health information about you that might be used or shared during this research**
  ☒ Information from your hospital/clinic records within this institution or elsewhere, that may be reasonably related to the conduct and oversight of the research study.

  ☒ New health information from tests, procedures, visits, interviews, or forms filled out as part of this research study.
- **Why health information about you might be used or shared with others**
  The reasons we might use or share your health information are:
  - To do the research described above
  - To make sure we do the research according to certain standards – standards set by ethics and law, and by quality groups

Participant ID:_______________

  - For public health and safety – for example, if we learn new health information that could mean harm to you or others, we may need to report this to a public health or a public safety authority
  - For treatment, payment, or health card operations

- **People and groups that may use or share your health information**

  1. People or groups within this institution

  ☒ Researchers and the staff involved in this research study

  ☒ Harvard review board that oversees the research

  ☐ Staff within this institution who need the information to do their jobs (such as billing, or for overseeing quality of care or research)

  2. People or groups outside the institution

  ☐ People or groups that we hire to do certain work for us, such as data storage companies, our insurers, or our lawyers

  ☐ Federal and state agencies such as the U.S. Department of Health and Human Services, the Food and Drug Administration, the National Institutes of Health, and/or the Office for Human Research Protection, and other U.S. or foreign government bodies, if required by law or involved in overseeing the research

  ☐ Organizations that made sure hospital/clinic standards are met

  ☐ The sponsor(s) of the research study, and people or groups it hires to help perform this research study

  ☒ Other researchers and medical centers that are part of this research study

  ☐ A group that oversees the data (study information) and safety of this research study

  ☐ Other:

- **Time period during which your health information might be used or shared with others**
  Because research is an ongoing process, we cannot give you an exact date when we will either destroy or stop using or sharing your health information.

Your Privacy Rights

- You have the right not to sign this form permitting us to use and share your private information for research. If you do not sign this form you cannot take part in this research study. This is because we need the private information of everyone who takes part.

- You have the right to withdraw your permission for us to use or share your private information for this research study. If you would like to withdraw your permission, you must notify the person in charge of this research study in writing.

- If you withdraw your permission, we will not be able to take back any information that has already been used or shared with others. This includes information used or shared to carry out the research study or to be sure the research is safe and of high quality.

- If you withdraw your permission you cannot continue to take part in this research study.

- You have the right to see and get a copy of your private information that is used or shared for treatment or for payment. To ask for this information, please contact the person in charge of this research study.
