# Supplementary material for: Protocol for a randomized controlled trial evaluating the impact of the Nurse-Family Partnership’s home visiting program in South Carolina on maternal and child health outcomes
Source: Trials. 2020 Dec 4;21:997. doi: 10.1186/s13063-020-04916-9 (PMC7716290; doi:10.1186/s13063-020-04916-9)
Supplement: Supplementary file 4 [file 13063_2020_4916_MOESM4_ESM.docx]

Additional Fille 4

RESOURCES FOR NEW MOMS AND BABIES: GREENVILLE COUNTY

Below is a list of services that may be available for you and your baby. For more help finding health care services, call (800) 868-0404 or visit www.scdhec.gov/Health/FamilyPlanning.

NATIONAL AND SOUTH CAROLINA

The CARE LINE
Toll-free helpline with information about health care for pregnant women, infants, and children.
855-472-3432

National Women's Health Information Center
Offers help answering questions about women's health issues and breastfeeding
1-800-994-9662
www.womenshealth.gov

South Carolina Primary Health Care Association
Information on locating a Community Health Center in your community
(803) 788-2778
www.scphca.org

United Way 211 Helpline
2-1-1 is a free and confidential 24/7 service that helps people across the U.S. find local resources.
Phone: Dial 211
www.211.org

Text4Baby
Text4Baby is a smartphone app that provides appointment reminders and educational information about pregnancy and child development. Free with participating wireless carriers.
www.text4baby.org

Postpartum Support International
Postpartum Support International provides a support hotline to women suffering from postpartum depression and links women to resources in their communities.
Phone: 1-800-944-4773
Text: 503-894-9453
www.postpartum.net
Facebook support group: Search "Postpartum Support International" on Facebook

Aunt Bertha
Aunt Bertha is a network of free and reduced-cost services throughout the country including medical care, food, housing, job training, and more.
www.auntbertha.org

Daniel's Law Safe Havens
Under South Carolina state law, a person can surrender an unharmed newborn baby up to 60 days after birth to a designated Safe Haven: hospitals, police, fire, and emergency medical services (EMS) stations, or a house of worship (only when these facilities are staffed). The baby will then be transferred to the custody of SC Department of Social Services (DSS).

National Domestic Violence Hotline
The National Domestic Violence Hotline is available to support and direct survivors of domestic violence to resources.
Phone: 1-800-799-7233
TTY: 1-800-787-3224
www.thehotline.org

GREENVILLE COUNTY

Mental Health Crisis Line of Greenville County
(864) 271-8888

Medicaid Transportation
Non-emergency transportation services to get to and from medical services
Logisticare – 1-866-910-7688

Greenville County Public Health Department
General information on healthcare, including family planning and clinics, as well as public benefits, such as Medicaid Optional Coverage for Women and Infants
Greenville Public Health Dept.
200 University Ridge
Greenville, SC 29601
(864) 282-4100
