# Supplementary material for: Protocol for a randomized controlled trial evaluating the impact of the Nurse-Family Partnership’s home visiting program in South Carolina on maternal and child health outcomes
Source: Trials. 2020 Dec 4;21:997. doi: 10.1186/s13063-020-04916-9 (PMC7716290; doi:10.1186/s13063-020-04916-9)
Supplement: Supplementary file 5 [file 13063_2020_4916_MOESM5_ESM.docx]

Additional File 5

Question	Answer
Welcome, NFP Nurse!<br><br>In order to enroll the client in the study today, you will need to enter her ETO case number.<br><br>If the client chooses to participate in the study, you'll also need to collect identifying information from her.<br><br>Please ask the client now if she has her Social Security Number and Medicaid ID number handy.	
Is this a practice form?	1 Yes<br>0 No
Select your site:	
Select your name:	
Will this client's visits be billed through MIECHV funding?	1 Yes<br>0 No<br>2 I'm not sure at this time. I will follow-up with my nurse supervisor.
Do you have the client's ETO case number at this time?	1 Yes<br>0 No
Please remember to submit a Missing ETO ID form on your tablet when this client's ETO case number becomes available.	
What is the ETO Case Number for this client?	
Please re-enter the ETO Case Number for this client to confirm.	
First, I'll ask you several questions to make sure that you are eligible.<br><br>If you are eligible, I can tell you more about the study and you can decide if you'd like to participate.<br>Please begin reading questions to the client.	
Based on the ETO ID, it appears that this client may have already been surveyed. Please double-check with the client if she has been surveyed in the past. Has this client been surveyed already?	
Are you currently pregnant?	1 Yes<br>0 No
This program is for women who are currently pregnant.<br>Please click the forward arrow to continue.	

Have you ever had a live birth?	1 Yes<br>0 No
This program is for first-time mothers. Please click the forward arrow to continue.	
When is your due date?<br>If unsure of exact date, best estimate is fine.	calendar
This program is for women who are 28 weeks or less into their pregnancy. Please click the forward arrow to continue.	
Are you currently enrolled in Medicaid?	1 Yes, I am enrolled in Medicaid<br>2 No, I meet Medicaid income criteria, but I am not a U.S. citizen or permanent resident (Green Card holder).<br>3 No, but I applied and my application is pending<br>4 No, my income is too high for Medicaid
This program is for Medicaid eligible families. Please click the forward arrow to continue.	
What county do you currently live in?	county list
This program is only for individuals belonging to the counties listed. Please click the forward arrow to continue.	
What is your date of birth?	calendar
Based on the date of birth and county this client listed, this client may have already been surveyed. Please double-check with the client that she has not already completed the survey. Has this client been surveyed already?	
Great - it seems like you're eligible to be part of the study!<br><br>I'm now going to review some information with you so that you can decide whether you'd like to participate or not. If you choose to participate, we'll ask you some survey questions, give you a $25 gift card to thank you for your time, and see if there's a spot available for you in NFP today.	

Statement of Consent<br><br>I have read the information, including risks and possible benefits, in the consent form. All of my questions regarding the research have been answered to my satisfaction. I understand that I am free to withdraw at any time without penalty or loss of benefits to which I am otherwise entitled.<br><br>I consent to participate in the study.	
Before we start the survey, I just want to remind you that your responses to these questions will in no way affect your chances of being selected for NFP. There are no right or wrong answers. Please just answer the questions as best you can!	
How did you hear about this project? If the client was referred by one of the 16 referral source codes listed in ETO, select “Referral Source Listed in ETO”.	1 Referral Source Listed in ETO (e.g. WIC, Pregnancy Testing Clinic)<br><br>2 Contacted from Medicaid referral list<br><br>3 Advertisement/Marketing Materials<br><br>4 Approached at Outreach Event<br><br>5 Approached at Referral Site (e.g. WIC site or Health Center)<br><br>-99 Other<br><br>-999 Refused to answer
1.0 What is the client's first name? Please enter her legal name, as it would appear on a legal document.	
1.1 What is the client's middle name? Leave blank if she does not have a middle name.	
1.2 What is the client's last name? Please enter her legal name, as it would appear on a legal document.	
1.3 Have you gone by any other name, including maiden names?	1 Yes 0 No
1.4 What is that name?	
2.0 How old are you?	

3.0 Do you know your Social Security Number?	1 Yes, I know my SSN. 2 I know only the last 4 digits. 3 Refused to answer. 4 I don't have one.
3.1 What is your Social Security Number?	
3.2 Please re-enter the 9 digit SSN to confirm.	
3.3 What are the last four digits of your SSN?	
3.4 Please re-enter the last four digits to confirm	
4.0 What is your Medicaid number? Please enter a 10 digit number, no dashes or other special punctuation.	
OR<br>1 Client cannot find Medicaid ID at this time.	
Please be sure to check again with your client.<br><br>To ensure that the client is eligible for the NFP program and that Medicaid will cover the NFP services delivered, it is important that the client's Medicaid ID is accurately recorded.	
If the client is now able to locate her ID please enter it here: Please enter a 10 digit number, no dashes or other special punctuation.	
4.1 What is your emergency Medicaid number?<br><br>If the client has an emergency Medicaid ID, please enter it below:<br>If this client does not have Emergency Medicaid, please press the forward button to continue.	
5.0 What city do you currently live in?	city list
5.1 What is your street address? Where you spend at least 4 nights per week. (Ex: 123 Flower Rd.)	
5.2 Unit or Apt Number:<br>(Ex: 2B)	
5.4 What is the zipcode for this address?	
5.5 Is this your complete and correct address?<br><br>If this address is not correct, please hit the survey back button to return to previous questions and edit any errors.	1 Yes 0 No
6.0 What is your primary phone number? (home or cell) Area code should be included for all 10 digit phone numbers.	

6.1 Do you have a second number? (home or cell) Leave blank if client does not have a second phone number.	
6.2 What is the name of your primary emergency contact?	
6.3 What is [q6_namcontact1]'s phone number? Please enter digits only, no dashes or special punctuation.	
6.4 How is [q6_namcontact1] related to you?	1 Relative 2 Friend 3 Co-worker 4 Neighbor 5 Partner 6 Spouse 7 Father of the child (FoC), not married 8 Other -999 Refused to answer
7.0 What is your ethnicity?	1 Hispanic or Latina 2 Not Hispanic or Latina 3 Declined to self-identify
8.0 What race(s) do you identify with? Check all that apply	1 American Indian or Alaska Native 2 Asian 3 Black or African-American 4 Native Hawaiian or other Pacific Islander 5 White 6 Declined to self-identify
9.0 Is any language other than English spoken in your home?	1 Yes 0 No -999 Refused to answer
10.0 What other languages are spoken in your home?	1 French (incl. Patois, Cajun) 2 Spanish 3 Cambodian (Khmer) 4 Tagalog 5 Chinese 6 Haitian Creole 7 German -99 Other -999 Refused to answer
10.1 Please specify this other language	
11.1 How well do you speak English? Would you say...	1 Very well 2 Well 3 Not very well 4 Not at all -999 Refused to answer
12.0 What is the highest grade or year of school that you have completed? Not the highest grade you have started, but the highest grade you have completed.	1 Grade 1 2 Grade 2 3 Grade 3 4 Grade 4 5 Grade 5 6 Grade 6 7 Grade 7 8 Grade 8 9 Grade 9 10 Grade 10 11 Grade 11 12 GED 13 High School Diploma 14 Vocational/certification/technical training program (beyond high school) 15 Associate Degree 16 BA/BS Degree 17 MA/Masters 18 PhD/Doctorate 19 Some college but no degree completion 20 No regular/formal school education -99 Other -999 Refused to answer
12.1 Please specify other	

13.0 Are you currently taking any education or training classes? This could include high school, ABE, GED or college courses, or any job skills training	1 Yes 0 No -999 Refused to answer
14.0 Are you currently serving in the military?	1 Yes 0 No -999 Refused to answer
15.0 How many times have you moved in the last 12 months?	0 Has not moved in past 12 months 1 1 time 2 2 times 3 3 times 4 4 times 5 5 times 6 6 times 7 7 times 8 8 times 9 9 times 10 10 times 11 Over 10 times -999 Refused to answer
15.1 What type of residence do you live in?	1 Private residence 2 Dormitory 3 Shelter/homeless -999 Refused to answer
15.2 With whom do you live? Check all that apply.	1 Live alone 2 A parent/parents 3 Father of the child (FoC) 4 Current partner/husband (not FoC) 5 Other family members 6 Other non-related individuals (roommates) -999 Refused to answer
16.0 What is your current marital status?	1 Married 2 Divorced 3 Separated 4 Never married 5 Widowed -999 Refused to answer
16.1 How often do you usually see or talk to the biological father?	1 Not at all 2 Less than once a week 3 At least once a week, but not daily 4 Daily -999 Refused to answer
17.0 Are you currently working for pay? (Include self-employment)	1 Yes 0 No -999 Refused to answer
17.2 What were your approximate total earnings from your work last month, including tips and overtime pay?<br><br>When answering, please include income from all jobs you held last month. Do NOT include earnings from anyone else within your household.<br><br>Best estimate is fine. (Before taxes).	Interger, OR -888 Don't Know, -999 Refused to answer
17.3 Last month, within which range were your earnings?	1 Less than $500 2 $500 to $999 3 $1,000 to $1,499 4 $1,500 to $1,999 5 $2,000 to $2,499 6 $2,500 to $2,999 7 $3,000 to $3,499 8 $3,500 or over -888 Don't know -999 Refused to answer

The next set of questions ask about your household. By household, I mean people who live together, usually eat together, and have common financial resources.<br><br>A household is often built around a family, but it doesn't have to be. If you are living with a partner and you generally pool your incomes, then please include their income here. But if you have roommates whose incomes and spending are mostly separate from yours, then please don't include them.	
17.4 Last month, what were the approximate total earnings of any other adult members in your household? Best estimate is fine.	Interger, OR -888 Don't Know, -999 Refused to answer
17.5 Last month, within which range were the earnings of other adult members of your household?	1 Less than $500 2 $500 to $999 3 $1,000 to $1,999 4 $2,000 to $2,999 5 $3,000 to $3,999 6 $4,000 to $4,999 7 $5,000 to $7,499 8 $7,500 to $9,999 9 $10,000 or over -888 Don't know -999 Refused to answer
Please tell me whether you or any members of your household received income or benefits from the following sources in the last month.	
18.0 Cash welfare, also known as TANF or Family Independence?	1 Yes 0 No -888 Don't know -999 Refused to answer
18.1 Food stamps or Supplemental Nutrition Assistance Program (SNAP) benefits?	1 Yes 0 No -888 Don't know -999 Refused to answer
18.2 Disability insurance such as Supplemental Security Income (SSI) or Social Security?	1 Yes 0 No -888 Don't know -999 Refused to answer
18.3 Unemployment benefits ?	1 Yes 0 No -888 Don't know -999 Refused to answer
18.4 WIC (the Special Supplemental Nutrition Program for Women, Infants, and Children)?	1 Yes 0 No -888 Don't know -999 Refused to answer
19.0 Do you currently have a bank account?	1 Yes 0 No -888 Don't know -999 Refused to answer
19.1 Does anyone in your household have a bank account?	1 Yes 0 No -888 Don't know -999 Refused to answer
19.2 Do you currently have a credit card?	1 Yes 0 No -888 Don't know -999 Refused to answer
19.3 Does anyone in your household have a credit card?	1 Yes 0 No -888 Don't know -999 Refused to answer
19.4 Do you currently have your own driver's license?	1 Yes 0 No -888 Don't know -999 Refused to answer
19.5 Does anyone else in your household have a driver's license?	1 Yes 0 No -888 Don't know -999 Refused to answer

19.6 Do you have access to a car?	1 Yes 0 No -888 Don't know -999 Refused to answer
In the past 12 months, since MM-DD-YYYY, please tell me whether the statements below were often true, sometimes true, or never true for your household:	
20.0 "We worried whether our food would run out before we got money to buy more."	1 Often true 2 Sometimes true 3 Never true -999 Refused to answer
21.0 "The food we bought just didn't last and we didn't have money to get more."	1 Often true 2 Sometimes true 3 Never true -999 Refused to answer
22.0 Are you covered by health insurance or some other kind of health care plan?	1 Yes 0 No -999 Refused to answer
23.0 What kind of health insurance or health care coverage do you have?<br><br>If you have more than one kind of health insurance, tell me about all the plans that you have<br><br>Include those that pay for only one type of service (e.g. dental care). If they are under parent's health coverage, ask what kind.<br><br>If the client answered that she has an "application pending" with Medicaid, she does not have Medicaid coverage yet and should NOT choose the Medicaid option below.	1 Private health insurance 2 Medicare 3 Medigap 4 Medicaid 5 SCHIP (Children's health insurance program) 6 Military health care (Tricare, VA, CHAMP-VA) 7 Indian Health Service 8 Single service plan (e.g. Dental, Vision, Prescriptions) -99 Other -999 Refused to answer -888 Don't know
23.1 Please specify this other plan:	
24.0 Is there a place you go to for general health care, if you are sick or need advice about your health?	1 Yes 0 No -888 Don't know -999 Refused to answer
24.1 What kind of place do you most frequently or usually go to for general health care?	1 Clinic 2 Health center 3 Doctor's office 4 Hospital/Emergency Room 5 Urgent Care 6 DHEC Health Department -99 Some other place -888 Don't know -999 Refused to answer
24.2 Please specify this other place:	
25.0 Is there a place you go, or have gone, for family planning or birth control?	1 Yes 0 No -888 Don't know -999 Refused to answer
25.1 What kind of place do you most frequently or usually go to for family planning or birth control?	1 Clinic 2 Health center 3 Doctor's office 4 Hospital/Emergency Room 5 Urgent Care 6 DHEC Health Department -99 Some other place -888 Don't know -999 Refused to answer
25.2 Please specify this other place:	

26.0 In the past 6 months, have you obtained care at the hospital emergency room for any reason?	1 Yes 0 No -999 Refused to answer
26.1 How many times have you obtained care at the hospital emergency room in the past 6 months?	
26.2 What was the purpose of these visits to the hospital emergency room?	1 Injury 2 Ingestion 3 Respiratory infection 4 Fever 5 Pain or headache 6 Vomiting, indigestion, or nausea 7 Vaginal bleeding 8 Chest pain 9 Abdominal pain 10 High blood pressure 11 Pregnancy-related -99 Other -999 Refused to answer
26.3 Please specify this other reason for the visit:	
27.0 In the past 6 months, have you obtained care at the urgent care center for any reason?	1 Yes 0 No -999 Refused to answer
27.1 How many times have you obtained care at the urgent care center in the past 6 months?	
27.2 What was the purpose of these visits to urgent care?	1 Injury 2 Ingestion 3 Respiratory infection 4 Fever 5 Pain or headache 6 Vomiting, indigestion, or nausea 7 Vaginal bleeding 8 Chest pain 9 Abdominal pain 10 High blood pressure 11 Pregnancy-related -99 Other -999 Refused to answer
27.3 Please specify this other reason for the visit:	
28.0 During the past year, have you received mental health help or treatment? Including anxiety, depresssion.	1 Yes 0 No -888 Don't know -999 Refused to answer
30.0 In general, before your current pregnancy, would you say your health was: Please read categories	1 Excellent 2 Very good 3 Good 4 Fair 5 Poor -888 Don't know -999 Refused to answer
31.0 How tall are you without shoes?	Feet and Inches, OR -888 Don't know, -999 Refused to answer
32.0 How much did you weigh before you were pregnant? You best estimate is fine.	
32.1 Please select the unit of the number above:	1 Pounds 2 Kilos -888 Don't know
33.0 Before you got pregnant, were you ever told by a doctor, nurse, or other health care worker that you had Type 1 or Type 2 diabetes? This is not the same as gestational diabetes or diabetes that starts during pregnancy.	1 Yes 0 No -888 Don't know -999 Refused to answer
34.0 Was this pregnancy planned?	1 Yes 0 No -888 Don't know -999 Refused to answer
34.1 Would you say you became pregnant too soon, at about the right time, or later than you wanted?	1 Too soon 2 Right Time 3 Later 4 Didn't care 5 Never wanted to have children -999 Refused to answer

35.0 Were you using any form of birth control when you got pregnant? Including: The pill (such as combined oral contraceptives or progestin only pills), barrier methods (such as male or female condoms, diaphragms, cervical caps), an IUD or implantable rod, the patch or a vaginal ring, injectables, spermicides or sponges, or the Rhythm/Calendar method.	1 Yes 0 No -888 Don't know -999 Refused to answer
36.0 What kind of birth control did you use? Select all that apply	1 The pill (such as combined oral contraceptives or progestin only pills) 2 Barrier methods (such as male or female condoms, diaphragms, cervical caps) 3 IUD or implantable rod 4 The patch or a vaginal ring 5 Injectables 6 Spermicides or sponges 7 Rhythm/Calendar method -999 Refused to answer
37.0 How many weeks or months pregnant were you when you had your first visit for prenatal care? A prenatal visit would include a regular check-up with a doctor, midwife, or other health worker for the treatment or prevention of potential health problems throughout the course of a pregnancy.<br><br>Do not count a visit that was only for a pregnancy test or only for WIC (the Special Supplemental Nutrition Program for Women, Infants, and Children).	Enter number of weeks, OR number of months, 88 Didn't receive any prenatal care, -999 Refused to answer
38.0 Did you want to get prenatal care sooner? If so, what kept you from getting prenatal care sooner? Select all that apply.	1 Nothing, I got it as soon as I wanted or needed to 2 I couldn't get an appointment when I wanted one 3 I didn't have enough money or insurance to pay for my visits 4 I had no transportation to get to the clinic or doctor's office 5 The doctor or my health plan would not start care as early as I wanted 6 I had too many other things going on 7 I couldn't take time off from work or school 8 I didn't have my medicaid card 9 I didn't know that I was pregnant 10 I didn't want prenatal care -99 Other -888 Don't know -999 Refused to answer
38.1 Please specify other reason:	
39.0 How many prenatal appointments have you had?	Interger, OR -888 Don't Know, -999 Refused to answer

40.0 During your pregnancy, have you been told by a doctor, nurse, or another health care worker that you have gestational diabetes (diabetes that started during this pregnancy)?	1 Yes 0 No -888 Don't know -999 Refused to answer
41.0 During your pregnancy, have you been told by a doctor, nurse, or other health care worker that you have high blood pressure, hypertension (including pregnancy-induced hypertension [PIH]), preeclampsia, or toxemia?	1 Yes 0 No -888 Don't know -999 Refused to answer
42.0 Do you intend to breastfeed?	1 Yes 0 No -888 Don't know -999 Refused to answer
43.0 Do you know if you are pregnant with:	1 One baby 2 Twins 3 Triplets 4 Quadruplets 5 More than quadruplets -888 I don't know yet -999 Refused to answer
43.1 Would you like to have more children one day?	1 Yes 0 No -888 Don't know -999 Refused to answer
44.0 How far apart in age do you want this child to be from your next child? If the client is having multiples, please ask:<br><br>How far apart in age do you want these children to be from your next child?<br><br>Looking for the desired age difference between this child and their next.	Interger, OR -888 Don't Know, -999 Refused to answer
44.1 How many children would you like to have overall?	Interger, OR -888 Don't Know, -999 Refused to answer
45.0 How often have you felt that you were unable to control the important things in your life?	0 Never 1 Almost never 2 Sometimes 3 Fairly often 4 Very Often -999 Refused to answer
45.1 How often have you felt confident about your ability to handle your personal problems?	0 Never 1 Almost never 2 Sometimes 3 Fairly often 4 Very Often -999 Refused to answer
45.2 How often have you felt that things were going your way?	0 Never 1 Almost never 2 Sometimes 3 Fairly often 4 Very Often -999 Refused to answer
45.3 How often have you felt difficulties were piling up so high that you could not overcome them?	0 Never 1 Almost never 2 Sometimes 3 Fairly often 4 Very Often -999 Refused to answer
Over the past two weeks, how often have you been bothered by any of the following problems?:	
46.0 Little interest or pleasure in doing things.	0 Not at all 1 Several days 2 More than half the days 3 Nearly every day -999 Refused to answer

47.0 Feeling down, depressed, or hopeless.	0 Not at all 1 Several days 2 More than half the days 3 Nearly every day -999 Refused to answer
48.0 Thinking about the last 6 months, could you have accessed the kinds of help listed below if you needed to?	1 Yes 0 No
48.1 Someone to loan you $50.	1 Yes 0 No
48.2 Someone to help you if you were sick and needed to be in bed.	1 Yes 0 No
48.3 Someone to take you to the clinic or doctor's office if you needed a ride.	1 Yes 0 No
48.4 Someone to talk with about your problems.	1 Yes 0 No
49.0 In the 3 months before you got pregnant, how many cigarettes did you smoke in an average week?<br><br>One pack is 20 cigarettes.<br><br>Enter 0 if respondent did not smoke at all.<br><br>Example: If client smoked one pack per day, enter 140 cigarettes. (7 days X 20 cigarettes)	Interger, OR -888 Don't Know, -999 Refused to answer
49.1 How many cigarettes did you smoke last week?<br><br>One pack is 20 cigarettes.<br><br>Enter 0 if respondent did not smoke at all.	Interger, OR -888 Don't Know, -999 Refused to answer
50.0 Which of the following statements best describes the rules about smoking inside your home now?	1 No one is allowed to smoke anywhere inside my home 2 Smoking is allowed in some rooms or at some times 3 Smoking is permitted anywhere inside my home -888 Don't know -999 Refused to answer
51.0 Have you been arrested within the past year?	1 Yes 0 No -888 Don't know -999 Refused to answer
52.0 During the 3 months before you got pregnant, how many alcoholic drinks did you have in an average week? By a "drink" we mean a can or bottle of beer, a wine cooler or glass of wine, a shot of liquor, or a mixed drink.	0 Didn't drink at that time 1 Less thank 1 drink 2 1 to 3 drinks 3 4 to 6 drinks 4 7 to 13 drinks 5 14 to 19 drinks 6 20 or more drinks -888 Don't know -999 Refused to answer
52.1 How many alcoholic drinks did you have last week? By a "drink" we mean a can or bottle of beer, a wine cooler or glass of wine, a shot of liquor, or a mixed drink.	0 Didn't drink at that time 1 Less thank 1 drink 2 1 to 3 drinks 3 4 to 6 drinks 4 7 to 13 drinks 5 14 to 19 drinks 6 20 or more drinks -888 Don't know -999 Refused to answer

53.0 Nurse: Did you use the English version or the Spanish version of the survey?	1 English 2 Spanish 3 Both
We've reached the end of the survey. Thanks for taking the time to answer those questions. I have a $25 Visa gift card for you. First, I need to take a picture of the front of the gift card.	
Now I need you to provide your signature to acknowledge that you received this gift card. By signing here, [q1_firstname] acknowledges that she has received a $25 Visa giftcard to compensate her for the time it took to complete this interview.	
Did you conduct the full survey?	1 Yes<br>0 No - Client refused consent.
We've reached the end of the interview. Thank you so much for answering these questions.<br><br>Now the computer will use a lottery to see whether there is a spot for you in the NFP program. It's like pulling a name out of a hat or flipping a coin, but the computer does it.<br><br>I have no control over who will get a spot and who won't. Everyone has the same chance of getting a spot in the program. The answers that you provided during the interview will not influence the outcome of the lottery.	
The tablet is processing the result. Please swipe forward and click the "Save Form and Exit" button to submit.<br><br>AFTER the survey has been submitted, you will be able to see the results of the lottery. Please be sure to record the result!	
[q1_firstname], there is a spot for you available in NFP. [surveyor_name], please remember to record this result in ETO within 48 hours.	
[q1_firstname], unfortunately there is no spot available for you in NFP. [surveyor_name], please remember to record this result in ETO within 48 hours.	
