# Supplementary material for: Protocol for a randomized controlled trial evaluating the impact of the Nurse-Family Partnership’s home visiting program in South Carolina on maternal and child health outcomes
Source: Trials. 2020 Dec 4;21:997. doi: 10.1186/s13063-020-04916-9 (PMC7716290; doi:10.1186/s13063-020-04916-9)
Supplement: Supplementary file 6 [file 13063_2020_4916_MOESM6_ESM.docx]

Additional File 6

HOME VISITOR SURVEY

A. DEMOGRAPHICS

1. What is the name of your agency?
    - ☐ Carolina Health Centers
    - ☐ DHEC Low Country
    - ☐ DHEC Midlands
    - ☐ DHEC Pee Dee
    - ☐ DHEC Upstate
    - ☐ Family Solutions Low Country
    - ☐ McLeod
    - ☐ Prisma Upstate
    - ☐ Spartanburg

2. What is your name?

3. How long have you been in your current role?
    - ☐ Less than 1 year
    - ☐ 1 year
    - ☐ 2 years
    - ☐ 3 years
    - ☐ 4 years
    - ☐ 5-9 years
    - ☐ 10 or more years

4. Please check all education completed:
    - ☐ Diploma Nursing
    - ☐ Associates Degree Nursing
    - ☐ Bachelor's Degree Nursing
    - ☐ Master's Degree Nursing
    - ☐ Associates Degree – other
    - ☐ Bachelor's Degree – other
    - ☐ Master's Degree – other
    - ☐ Professional Degree (e.g. LLB, LD, MD)
    - ☐ ND (Nursing Doctorate) or DNP (Doctorate of Nursing Practice)
    - ☐ PhD in Nursing or DNS (Doctor of Nursing Science)
    - ☐ Doctorate Degree – other

5. Did you have any previous experience with NFP before becoming an NFP home visiting nurse or nurse supervisor? Check all that apply.
    - ☐ I was an NFP mom
    - ☐ A family member, friend or colleague was an NFP mom
    - ☐ A family member, friend or colleague worked for NFP
    - ☐ No direct experience with NFP, but had heard about NFP
    - ☐ Never heard of NFP before applying for this role

6. How long have you lived in South Carolina?
   - ☐ Less than a year
   - ☐ 1-2 years
   - ☐ 3-5 years
   - ☐ 5-10 years
   - ☐ More than 10 years

7. Prior to your current housing, did you ever live in one of the counties currently served by your agency?
   - ☐ Yes
   - ☐ No

8. Do you currently live in one of the counties currently served by your agency?
   - ☐ Yes
   - ☐ No

9. What is your ethnicity?
   - ☐ Hispanic or Latina
   - ☐ Not Hispanic or Latina
   - ☐ Declined to self-identify

10. What is your race? One or more categories may be selected.
    - ☐ White
    - ☐ Black or African American
    - ☐ American Indian or Alaska Native
    - ☐ Asian
    - ☐ Native Hawaiian or other Pacific Islander
    - ☐ Declined to self-identify

11. What is your age?
    - ☐ Under 20 years
    - ☐ 20-29
    - ☐ 30-39
    - ☐ 40-49
    - ☐ 50-59
    - ☐ 60 or older

12. In addition to your job at NFP, are you doing any other work for pay?
    - ☐ No
    - ☐ Yes, please describe:

13. Are you currently pursuing a higher degree or taking continuing education classes?
    - ☐ No
    - ☐ Yes, please describe the classes you are taking and the degree you are pursuing (if applicable):

B. EMPLOYMENT HISTORY

1. Prior to joining NFP, how many years of nursing practice experience do you have?

2. Prior to joining NFP, did you have any RN experience in the following settings/environments? Check all that apply.

   - ☐ Medical and/or surgical unit
   - ☐ Critical or intensive care
   - ☐ Community health
   - ☐ Adolescent health
   - ☐ Pediatrics
   - ☐ Mental health
   - ☐ Women's health/ OBGYN
   - ☐ Long term care/ nursing home
   - ☐ Home visiting
   - ☐ School health
   - ☐ Other, please specify:
   - ☐ I do not have any nursing experience prior to joining NFP.

C. CURRENT POSITION

1. Over the **past month**, how many hours did you work in a typical week?

   HOURS: ___________

2. Thinking back to **last week**, please enter the number of hours you spend doing each of the following activities. You can enter part of an hour for an activity that is less than an hour. Please enter 0 for activities you did not do.

Home visiting (excluding first visits)	___________
Study enrollment (eligibility assessment, informed consent, baseline survey)	___________
Recruiting families	___________
Preparing for home visits (excluding study visits)	___________
Travel to home visits	___________
Transporting families	___________

Telehealth	
Staff group meetings	
NFP Paperwork	
Data entry into computer	
Receiving supervision	
Training	
Grant writing<br>Providing supervision and professional development<br>Recruiting/hiring/training staff	
Other (specify hours):	

3. Please express your agreement or disagreement with the following statement: I always feel comfortable meeting clients in their home.
   - ☐ Strongly Disagree
   - ☐ Disagree
   - ☐ Agree
   - ☐ Strongly Agree

These next 2 questions ask about how you feel about your job at the moment. Please remember that the research team follows strict rules to keep your information private. No reports will include your name or other identifying information. And no identifying information will be shared with anyone outside of the research team, including NSO.

4. Taking everything into consideration, how satisfied do you feel with your job as a whole?

1	2	3	4	5	6	7
Not at all satisfied			Moderately satisfied			Extremely satisfied

5. Overall, based on your definition of burnout, how would you rate your level of burnout?
   - ☐ I enjoy my work, I have no symptoms of burnout.
   - ☐ Occasionally I am under stress, and I don't always have as much energy as I once did, but I don't feel burned out.
   - ☐ I am definitely burning out and have one or more symptoms of burnout, such as physical and emotional exhaustion.
   - ☐ The symptoms of burnout that I'm experiencing won't go away. I think about frustration at work a lot.
   - ☐ I feel completely burned out and often wonder if I can go on. I am at the point where I may need some changes or may need to seek some sort of help.

D. SERVICES PROVIDED

1. In what language(s) are you fluent enough to provide home visiting services? CHECK ALL THAT APPLY.

☐ English
☐ Spanish
☐ Other (specify): __________

2. How many families are in your current caseload? __________

3. Please rate the size of your current caseload:

☐ Lighter than you are able to handle
☐ About right
☐ Heavier than you are able to handle

4. In the past 6 months, how often have you had a caseload that was more than what you could handle effectively?

☐ All of the time
☐ Most of the time
☐ About half of the time
☐ Some of the time
☐ None of the time

5. Thinking about home visits from last week, how often did you prepare home visit plans in advance of these visits?

☐ All of the time
☐ Most of the time
☐ About half of the time
☐ Some of the time
☐ None of the time

6.Among the clients you've seen in the past 6 months, how often was the mother's primary care, OB/GYN, or other medical provider aware that she was in a home visiting program?

☐ All of my clients
☐ Most of my clients
☐ Some of my clients
☐ Few of my clients
☐ None of my clients

7. In the past 6 months, how often have you communicated with an expectant mother's primary care provider, OB/GYN, or other medical provider?

☐ Never
☐ Rarely during a client's pregnancy
☐ At least once during a client's pregnancy
☐ A few times during a client's pregnancy

- ☐ Regularly during a client's pregnancy

8. In the past 6 months, how often have you been able to view a paper or electronic copy of an expectant mother's medical record from her primary care provider or OB/GYN? (if never skip to question 8)
    - ☐ Never
    - ☐ Sometimes
    - ☐ Usually
    - ☐ Always

9. In the past 6 months, how have you used the information from the medical record in your home visits? (Check all that apply)
    - ☐ I don't really use it
    - ☐ I use it to plan for my upcoming home visits
    - ☐ I use it to follow up on recommendations made by the provider
    - ☐ I use it to review results of tests with the expectant mother
    - ☐ I use it to make sure that she is receiving necessary prenatal care
    - ☐ Other: ____

E. PROGRAM OUTCOMES

In this section, we would like to learn how *staff members* perceive their program's intended outcomes. In general, a *program outcome* is a benefit to a child, parent, or family. For example, some programs might see the improvement of prenatal health as an important outcome.

Below is a list of possible outcomes for home visiting programs. We know your program may care about all of these benefits for your families. However, we would like to get a sense of which outcomes you think **your program** believes may be more important than others. We would like you to check the box that best represents what you think your program believes about the outcome.

To help you decide on an outcome's rank, think about whether it has been routinely discussed in training and supervision over the past 6 months. Think about what staff in your agency have been told about its importance over the past 6 months. Check the box that best describes your program's ranking of this outcome.

1. Considering all of the outcomes your program aims to achieve, how much of a priority is <u>promoting good prenatal health</u>, such as diet, exercise, rest, and not smoking?

☐	☐	☐	☐	☐	☐	☐	☐	☐	☐	☐
0	1	2	3	4	5	6	7	8	9	10
Not a Priority at All					Moderate Priority					Highest Priority

2. Considering all of the outcomes your program aims to achieve, how much of a priority is <u>preventing poor birth outcomes</u>, such as pre-term birth and low birth weight?

☐	☐	☐	☐	☐	☐	☐	☐	☐	☐	☐
0 Not a Priority at All	1	2	3	4	5 Moderate Priority	6	7	8	9	10 Highest Priority

3. Considering all of the outcomes your program aims to achieve, how much of a priority is <u>promoting breastfeeding</u>?

☐	☐	☐	☐	☐	☐	☐	☐	☐	☐	☐
0 Not a Priority at All	1	2	3	4	5 Moderate Priority	6	7	8	9	10 Highest Priority

4. Considering all of the outcomes your program aims to achieve, how much of a priority is <u>promoting maternal physical health outside of pregnancy</u>, such as good nutrition, exercise, and rest?

☐	☐	☐	☐	☐	☐	☐	☐	☐	☐	☐
0 Not a Priority at All	1	2	3	4	5 Moderate Priority	6	7	8	9	10 Highest Priority

5. Considering all of the outcomes your program aims to achieve, how much of a priority is <u>promoting family planning and birth spacing</u>?

☐	☐	☐	☐	☐	☐	☐	☐	☐	☐	☐
0 Not a Priority at All	1	2	3	4	5 Moderate Priority	6	7	8	9	10 Highest Priority

6. Considering all of the outcomes your program aims to achieve, how much of a priority is <u>preventing and reducing maternal tobacco use</u>?

☐	☐	☐	☐	☐	☐	☐	☐	☐	☐	☐
0 Not a Priority at All	1	2	3	4	5 Moderate Priority	6	7	8	9	10 Highest Priority

7. Considering all of the outcomes your program aims to achieve, how much of a priority is <u>preventing and reducing maternal mental health and substance use problems</u>?

☐	☐	☐	☐	☐	☐	☐	☐	☐	☐	☐
0<br>Not a Priority at All	1	2	3	4	5<br>Moderate Priority	6	7	8	9	10<br>Highest Priority

8. Considering all of the outcomes your program aims to achieve, how much of a priority is <u>preventing and reducing domestic violence</u>?

☐	☐	☐	☐	☐	☐	☐	☐	☐	☐	☐
0<br>Not a Priority at All	1	2	3	4	5<br>Moderate Priority	6	7	8	9	10<br>Highest Priority

9. Considering all of the outcomes your program aims to achieve, how much of a priority is <u>promoting family economic self-sufficiency</u>, such as reaching goals for employment and education?

☐	☐	☐	☐	☐	☐	☐	☐	☐	☐	☐
0<br>Not a Priority at All	1	2	3	4	5<br>Moderate Priority	6	7	8	9	10<br>Highest Priority

10. Considering all of the outcomes your program aims to achieve, how much of a priority is <u>promoting children's preventive health care</u>, such as having all recommended well-child visits, being up-to-date on immunizations, and having parents baby-proof their home to prevent injuries?

☐	☐	☐	☐	☐	☐	☐	☐	☐	☐	☐
0<br>Not a Priority at All	1	2	3	4	5<br>Moderate Priority	6	7	8	9	10<br>Highest Priority

11. Considering all of the outcomes your program aims to achieve, how much of a priority is <u>promoting positive parenting behaviors</u>, such as nurturing, encouraging the child's learning, and using positive behavior management techniques?

☐	☐	☐	☐	☐	☐	☐	☐	☐	☐	☐
0<br>Not a Priority at All	1	2	3	4	5<br>Moderate Priority	6	7	8	9	10<br>Highest Priority

12. Considering all of the outcomes your program aims to achieve, how much of a priority is preventing and reducing child abuse and neglect?

☐	☐	☐	☐	☐	☐	☐	☐	☐	☐	☐
0<br>Not a Priority at All	1	2	3	4	5<br>Moderate Priority	6	7	8	9	10<br>Highest Priority

13. Considering all of the outcomes your program aims to achieve, how much of a priority is promoting child cognitive and language development and social- emotional well-being?

☐	☐	☐	☐	☐	☐	☐	☐	☐	☐	☐
0<br>Not a Priority at All	1	2	3	4	5<br>Moderate Priority	6	7	8	9	10<br>Highest Priority

13. Considering all of the outcomes your program aims to achieve, how much of a priority is coordinating services for families (i.e. referrals to services such as WIC, housing assistance, public assistance, education, substance abuse, and others)?

☐	☐	☐	☐	☐	☐	☐	☐	☐	☐	☐
0<br>Not a Priority at All	1	2	3	4	5<br>Moderate Priority	6	7	8	9	10<br>Highest Priority

F. SERVICE REFERRALS AND COORDINATION

The following questions are about the services that are available in your area and service coordination.

Services:

Prenatal Care
Maternal Postpartum Care / Care Related to Chronic Conditions for Mother (hypertension, diabetes)
Primary care provider – Child
Lactation support
Family Planning and Reproductive Health Care
Smoking Cessation or Substance Use (Alcohol and other drugs) Treatment
Mental Health treatment or therapy

Housing assistance/ Eviction prevention/ Homeless assistance
Medicaid
WIC
SNAP
TANF / DSS Family Independence program
Adult Education Services (including GED, ESL, and higher education)
Job Training and Employment
Childcare services and assistance/ daycare / ABC voucher
Early intervention services / head start
Child birth or parenting education or resources
Child Injury Prevention (like car seats, cribs, shaking baby prevention, safe sleep)

1. In the past 6 months, have you referred to a specific provider/agency that offers [SERVICE TYPE] ?

- ☐ Yes
- ☐ No [skip to next service]
- ☐ N/A – no service available [skip to next service]

2. Overall, how effective do you think this provider/agency has been in meeting families' needs for [SERVICE TYPE]?

- ☐ Very effective
- ☐ Quite effective
- ☐ Somewhat effective
- ☐ Not effective at all
- ☐ Unsure

3. Overall, how would you rate how well you and this provider/agency are able to share information about the families you refer?

- ☐ Excellent
- ☐ Good
- ☐ Fair
- ☐ Poor
- ☐ Unsure

4.Are there any other services or programs not mentioned above that you have referred mothers and their families to in the past 6 months? Please describe:

For the following questions, please note: By "referring and coordinating services", we mean all of the activities involved in connecting families to programs and services including discussing services with client, preparing applications, contacting agencies, scheduling appointments, following-up with client, etc.

5. Thinking about the past 6 months, how much time do you usually spend with clients *during a typical visit* on referring/coordinating services)?

☐ Nearly all of a visit (more than 90% of the time)
☐ Most of a visit (60-90% of the time)
☐ About half of a visit (40-60% of the time)
☐ Less than half of a visit (10-40% of the time)
☐ None or almost no time spent (0-10% of the time)

6.Please express your agreement or disagreement with the following statement: My clients really benefit from the time I spend helping them in referring and coordinating services.

☐ Strongly Agree
☐ Agree
☐ Disagree
☐ Strongly disagree

7. Please express your agreement or disagreement with the following statement: My time with a client would be better spent on other home visiting activities rather than on referring and coordinating services.

☐ Strongly Agree
☐ Agree
☐ Disagree
☐ Strongly disagree

G. CONCERNS

Please express your agreement or disagreement with the statements below.

**I am sometimes concerned it could hurt my relationship with a mother if I talk with her about....**	**Strongly Agree**	**Agree**	**Slightly Agree**	**Neutral**	**Slightly Disagree**	**Disagree**	**Strongly Disagree**
1. Prenatal nutrition, exercise, and access to care.	☐	☐	☐	☐	☐	☐	☐

I am sometimes concerned it could hurt my relationship with a mother if I talk with her about….	Strongly Agree	Agree	Slightly Agree	Neutral	Slightly Disagree	Disagree	Strongly Disagree
2. Her prenatal care provider's recommendations.	☐	☐	☐	☐	☐	☐	☐
3. Her physical health habits and access to primary care outside of pregnancy.	☐	☐	☐	☐	☐	☐	☐
4. Her family planning and birth spacing.	☐	☐	☐	☐	☐	☐	☐
5. Her tobacco use.	☐	☐	☐	☐	☐	☐	☐
6. Her alcohol and other drug use.	☐	☐	☐	☐	☐	☐	☐
7. Her mental health.	☐	☐	☐	☐	☐	☐	☐
8. Her relationships with family and friends.	☐	☐	☐	☐	☐	☐	☐
9. Partner violence.	☐	☐	☐	☐	☐	☐	☐
10. Her plans for school and work	☐	☐	☐	☐	☐	☐	☐
11. The public benefits she receives and needs.	☐	☐	☐	☐	☐	☐	☐
12. Breastfeeding.	☐	☐	☐	☐	☐	☐	☐
13. How she manages her child's behavior.	☐	☐	☐	☐	☐	☐	☐
14. Her child's development.	☐	☐	☐	☐	☐	☐	☐
15. Home safety.	☐	☐	☐	☐	☐	☐	☐
16. Her child's health care.	☐	☐	☐	☐	☐	☐	☐
17. Her child care arrangements.	☐	☐	☐	☐	☐	☐	☐

H. IMPACT

Please express your agreement or disagreement with the statements below.

I feel that as a result of the services I have provided....	Strongly Agree	Agree	Slightly Agree	Neutral	Slightly Disagree	Disagree	Strongly Disagree
18. More mothers support their children's social-emotional development.	☐	☐	☐	☐	☐	☐	☐
19. Children have better cognitive and language development.	☐	☐	☐	☐	☐	☐	☐
20. More children are securely attached.	☐	☐	☐	☐	☐	☐	☐
21. Fewer children are abused or neglected.	☐	☐	☐	☐	☐	☐	☐
22. Fewer homes have safety hazards.	☐	☐	☐	☐	☐	☐	☐
23. More children are up to date on their shots and well child care.	☐	☐	☐	☐	☐	☐	☐
24. Fewer children have injuries requiring medical care.	☐	☐	☐	☐	☐	☐	☐

2. What would help you better serve your families? Please include anything that seems relevant (i.e. education, training, supervision, mentorship, resources, compensation).

I. SUPERVISION

1. Thinking about the past 6 months, how often have you had one-on-one supervision meetings with your supervisor?

   ☐ Weekly or more frequently
   ☐ Every two weeks
   ☐ Every three weeks
   ☐ Monthly
   ☐ Once every 1-3 months
   ☐ Once every 4-6 months
   ☐ Never

2. Thinking about the past 6 months, how often have you had group supervision meetings with your supervisor?

   ☐ Once a week or more
   ☐ Every two weeks
   ☐ Every three weeks
   ☐ Once a month
   ☐ Once every 1-3 months
   ☐ Once every 4-6 months
   ☐ Never

Thank you for completing the survey! Please provide your name and email address so we can deli
your $25 gift card.

If you have any questions about the survey, please contact our study team at *<< email omitted in publication>>* or at *<< phone number omitted for publication>>*
